# Mediterranean diet component oleic acid decreases systemic impact of periodontal *Porphyromonas gingivalis*-infection in age: addressing role of stress resistance and microbiome

**DOI:** 10.1038/s41514-025-00248-7

**Published:** 2025-06-28

**Authors:** Annika Döding, Ludwig Wurschi, Cristina Zubiria-Barrera, Tilman E. Klassert, Leonhard Bereuter, Zhigang Rao, Ann-Kathrin Bär, Anton Domuncu, Andreas Koeberle, Heidi Noels, Hortense Slevogt, Bernd Sigusch, Ulrike Schulze-Späte

**Affiliations:** 1 Section of Geriodontics, Department of Conservative Dentistry and Periodontology, Center of Dental Medicine, University Hospital Jena, Jena, Germany; 2 Respiratory Infection Dynamics, Helmholtz Centre for Infection Research—HZI Braunschweig, Braunschweig, Germany; 3 Department of Respiratory Medicine and Infectious Diseases, Hannover Medical School, German Center for Lung Research (DZL), BREATH, Hannover, Germany; 4 Michael Popp Institute and Center for Molecular Biosciences Innsbruck (CMBI), University of Innsbruck, Innsbruck, Austria; 5 Institute of Pharmaceutical Sciences and Excellence Field BioHealth, University of Graz, Graz, Austria; 6 Institute for Molecular Cardiovascular Research (IMCAR), Uniklinik RWTH Aachen, RWTH Aachen University, Aachen, Germany; 7 Department of Conservative Dentistry and Periodontology, Center of Dental Medicine, University Hospital Jena, Jena, Germany

**Keywords:** Microbiology, Physiology, Diseases, Health care, Medical research, Pathogenesis, Risk factors

## Abstract

Periodontitis (PD) is an age-associated infectious periodontal disease driven by dysbiosis and hyperinflammatory host response, resulting in bone and tissue loss. Often linked to systemic inflammatory comorbidities, modulating host response emerged as promising clinical target. This study investigates whether nutritional intervention mitigates age-associated systemic impact of oral infection with PD key pathogen *Porphyromonas gingivalis*. Young (5 weeks) and aged (≥73 weeks) mice received palmitic acid-enriched Western-diet (PA-ED) or Mediterranean-diet enriched with oleic acid (OA-ED), both known for immunomodulatory properties. PA-ED exacerbated oral bone loss and systemic effects, particularly in aged mice, correlated with gut microbiota destabilization and impaired stress responses. Further, PA-ED enhanced osteoclast differentiation and primed bone marrow cells towards inflammation. Contrarily, OA-ED mitigated these effects. Osteoblasts showed baseline inflammation and reduced responsiveness in aged mice, thereby promoting a pro-inflammatory micro-environment. Findings suggest OA-ED as protective, especially for the elderly, by limiting local and systemic tissue damage associated with PD.

## Introduction

Age is the most powerful risk factor for many human diseases such as cardiovascular disease, diabetes or dementia^1^. This increased prevalence is not only due to a reduced regenerative capacity but also to changes in the immune system resulting in increased basal inflammation (‘inflammaging’) and impaired reactivity (‘immunosenescence’)^2,3^.

Periodontitis, associated with chronic diseases such as type 2 diabetes mellitus or cardiovascular disease, is another major age-related disease whose manifestation is driven by a hyperinflammatory immune response, pathological changes in the oral microbiome and systemic inflammation. The global prevalence of severe periodontal diseases (PD) is already at 19% of the global adult population, representing more than 1 billion cases worldwide. It is expected to increase in the coming years due to the demographic development with a growth in the aging population^4,5^. PD is a disease of the tissues enclosing the teeth, where plaque accumulation and the establishment of bacterial key pathogens as part of an oral dysbiosis cause an escalated inflammatory host response and disturbed resolution of inflammation, leading to damage in periodontal tissue and resulting in the loss of alveolar bone and teeth^4,6–8^. Besides local oral effects of PD, mediators of non-resolving inflammatory pathways as well as causative bacteria and bacterial products can spread systemically and compromise systemic tissue homeostasis^4^. Thereby, in the presence of comorbidities such as cardiovascular disease, diabetes, osteoporosis and others, tissue homeostasis can be compromised distant from the site of infection and increase systemic bone loss^4,9,10^. Subsequently, novel supportive treatments focus on immunomodulation to reduce tissue damage and recent human and animal studies underlined nutrition as a potent immunometabolic intervention^6,11,12^.

With the increased focus on plant-based foods in a Mediterranean-style diet (MD), Western-style diets (WD) and MD differ significantly in their fatty acid profiles. Thereby, the monounsaturated fatty acid (FA) oleic acid (OA (C18:1)), main component of MD and a major component of olive oil, is generally the most common unsaturated FA in human nutrition and serum^4,6^. OA-serum levels correlate negatively with periodontal tissue loss in patients following an optimized anti-inflammatory diet^12,13^. In contrast, levels of saturated FAs, specifically the WD-component palmitic acid (PA (C16:0)), correlate positively with periodontal disease (PD)^14^. In a murine model of periodontal infection with key pathogen *Porphyromonas gingivalis* (*P. gingivalis*), an OA-enriched diet compared to mice on isocaloric WD-style PA-enriched diet, resulted in lower systemic inflammation and reduced alveolar bone loss under obese conditions and improved femoral bone microarchitecture in normal-weight mice^4,15^. The damaging effect of PA is mediated by lipotoxic metabolites such as diacylglycerols (DAGs) and ceramides, which are associated with apoptosis, ER stress and IL-6-mediated inflammation^4^. Ultimately, this increases differentiation and activity of bone-resorbing osteoclasts and hinders mineralization activity and differentiation of bone-forming osteoblasts^16,17^, resulting in bone loss. Substituting PA with OA leads to the conversion of DAGs into triglycerides. Moreover, enhanced OA-intake increases levels of inflammation-resolving mediators^6,18^ and stress resistance-mediating phosphatidylinositols (PI)^4,19^; resulting in attenuation of bacterially-induced alveolar and femoral bone loss^4,6^.

Periodontitis-associated bacteria can also enter the gastrointestinal tract and negatively affect intestinal eubiosis^8,20,21^. Thus, associations between oral and gut dysbiosis in severe forms of periodontitis have already been described^22^. Furthermore, nutritional intake and ingested dietary fats directly influence the intestinal microbiome by altering its taxonomic and phylogenetic composition^23–25^. A subsequent dysbiosis could shape susceptibility to diseases such as cardiovascular diseases or diabetes^26–31^. Complex signaling pathways and stress-regulating interactions such as epigenetic changes in host cells^32^, production of inflammation-modulating short-chain fatty acids (SCFAs)^32^*, Bacteroidetes*-mediated biosynthesis of PI^33^ affecting barrier integrity^34^, and modulation of Stearoyl-CoA desaturase 1 (SCD-1) activity - a key enzyme in lipid metabolism and stress resilience^19,35,36^ form the basis of its immune-modulatory function affecting body physiology and tissue homeostasis.

Various studies, both human and murine, linked the stability of the gut microbiome to bone health^37^. Patients with low bone mineral density (BMD) show a decrease in the ratio between the two main phyla, *Firmicutes* and *Bacteroidetes*^26^. However, species of the intestinal microbiome exerts complex and non-uniform effects on bone metabolism in different areas of the body, as seen with *Lachnospiraceae*: while this family is associated with improved BMD in individuals suffering from osteoporosis^26^, they are elevated in periodontitis (PD)^20^. Nevertheless, its immunomodulatory function might be affected by age since the gut microbiome undergoes compositional changes and its diversity declines with age^38–40^. Though, there is evidence for both - an age-associated increase and decrease in *Firmicutes/Bacteroidetes* (*F/B*) ratio at phylum level and therefore an exact composition is still uncertain^39–41^.

Host-response driven diseases such as periodontitis and its disseminating infection become more prevalent in old age, which causes an increased economic burden^10^. Therefore, individualized periodontal patient care targeting modifiable risk factors such nutritional intake is increasingly important and of clinical relevance for the aging population - specifically, since Western-style diets with its high level of saturated fats are very common. Notably, a diet enriched with saturated fats is associated with PD progression in the elderly^42^. Still, regulatory pathways as a basis for preventive algorithms need to be determined. Whether an aged organism could benefit from specific nutritional components or substitution is not known. Based on its potent immunomodulatory function, we determined whether nutritional interventions with dietary OA as compared to WD-style component PA could modulate response to periodontal bacterial stimuli and protect from systemic impact of periodontal *P. gingivalis*-infection in age. To that extent, homeostasis of the local oral bone microenvironment, stability of systemic bone and resilience of the distant immune-regulatory microbiome in aged organisms were investigated.

## Results

### Palmitic acid-enriched diet increases oral bone loss in response to periodontal *Porphyromonas gingivalis*-infection

**Fig. 1:**
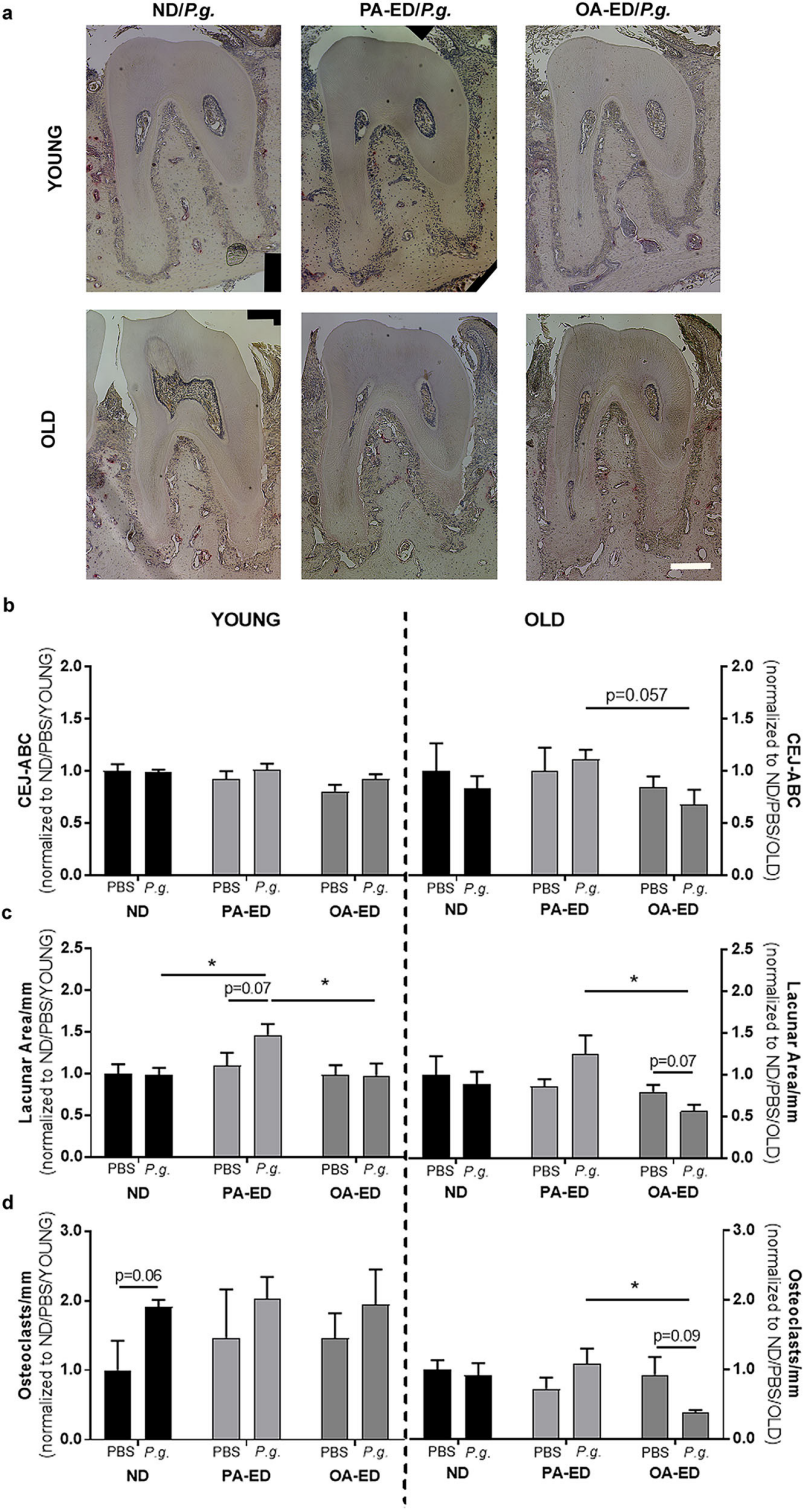
Combined palmitic acid intake and age exacerbate the bone-destructive impact of *P. gingivalis.* Young (5 weeks) and old (≥73 weeks) mice were fed either normal (ND), palmitic acid (PA) enriched diets (ED) or oleic acid (OA) ED inoculated with *Porphyromonas gingivalis* (*P.g*.) or PBS as control to induce periodontitis. **a** TRAP-stained mandibular bone sections of young (upper panel) and old (lower panel) *P.gingivalis*-infected animals after 16 weeks of dietary intervention. Sections were stained for TRAP (depicted in red) and counterstained with Meyer’s Hemalaun (blue). **b** Crestal bone loss was analyzed as the CEJ–ABC distance measured at the proximal bone peak between M1 and M2. **c** Number of TRAP^+^ osteoclasts (OC) per millimeter (mm). **d** Lacunae in alveolar bone adjacent to PdL. Data sets of the two age groups (young vs old) shown in (**b–d)** were depicted as fold change of ND/PBS within their respective age group. Data derived from 5 to 6 mice per condition (ND - normal standard diet, PA - PA-ED, OA- OA-ED; ctrl, placebo-infection; *P.g.* - *Porphyromonas gingivalis* infection). Statistical analysis: ANOVA with post hoc test (Tukey), within treatment groups, and two-tailed Student’s *t* test for pairwise comparisons. *p < 0.05. **Scale bar in** (**a**)**: 200** **μm**.

To investigate whether nutritional substitution with oleic acids as opposed to palmitic acid protects from alveolar bone loss in periodontally infected aged animals, young (5 weeks) and old (73 weeks) C57BL5JRj mice received identical diets either supplemented with palmitic acid (PA)- or oleic acid (OA-ED) for a total of 16 weeks and compared to a normal standard diet (ND). After five weeks of oral inoculation with *P. gingivalis*, histomorphometric analyses of mandibular bone (Figs. S1 and 1a) revealed no changes in alveolar bone crest height in young animals (Fig. 1b, left), whereas distance between cemento-enamel junction and alveolar bone crest (CEJ-ABC) increased by approximately 63% in old *P. gingivalis*-infected PA-ED animals as compared to aged animals fed an OA-ED (*p* = 0.057) (Fig. 1b, right). In deeper regions of the jawbone, *P .gingivalis*-inoculation increased bone loss surrounding the periodontal ligament (PDL) in young and old animals on PA-ED as compared to other nutritional concepts (YOUNG: PA-ED/*P.g.* vs ND/*P.g.*: *(*p* < 0.05), PA-ED/*P.g*. vs OA-ED/*P.g.:* *(*p* < 0.05), OLD: PA-ED/*P.g*. vs OA-ED/*P.g*.: *(*p* < 0.05) (Fig. 1a–c)). Furthermore, bone loss in the PDL area was accompanied by enhanced osteoclast numbers in aged PA-ED/*P.g.* as compared to aged OA-ED/*P.g.* groups (OLD: PA-ED/P.g. vs OA-ED/*P.g.*: *(*p* < 0.05) (Fig. 1d)). Overall, *P. gingivalis* infection manifests itself especially diet-dependently, which is particularly evident in aged animals.

### Dietary modulation changes the microbiome rapidly and permanently

**Fig. 2:**
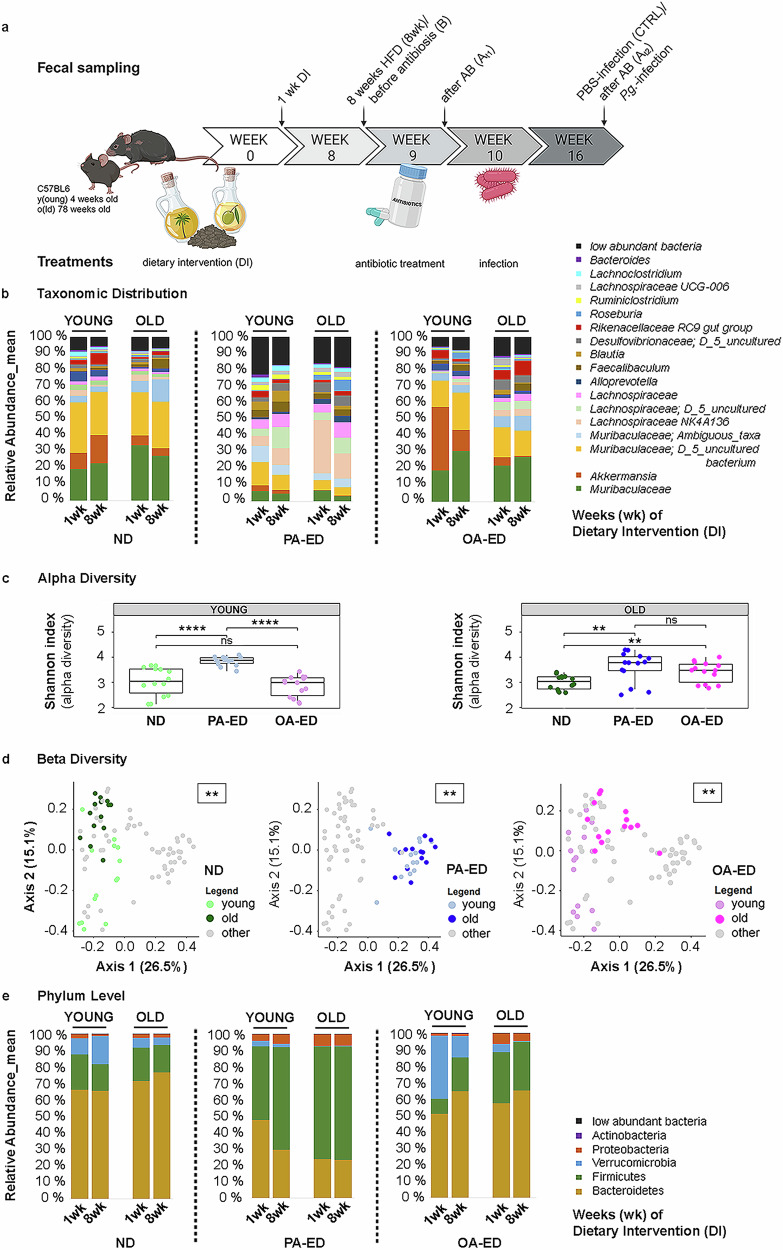
Gut microbiome is rapidly and persistently modulated by diet with age-dependent differences in beta-diversity. **a** Young (5 weeks) and old (≥73 weeks) mice received dietary interventions (DI) with either normal (ND), palmitic acid (PA) enriched diets (ED) or oleic acid (OA) ED for a total of 16 weeks. Animals received antibiotics for 5 d in week 9. Starting in week 10, they were either inoculated with *Porphyromonas gingivalis* (*P.g*.) or PBS as control three times a week for a total of 5 weeks. Animals were sacrificed in week 16. Microbiome samples were collected at four time points: after 1 week of dietary intervention (DI) (1wk), after 8 weeks of DI/before antibiosis (8wk/B), after antibiosis (At_1_), after infection (uninfected also At_2_). **b–e** Microbiome analyses of stool samples after 1 and 8 weeks of DI. **b** Taxonomic summary of bacterial compositional changes across age (‘young’, ‘old’) and nutrition (ND, PA-ED, OA-ED)-groups. **c** Samples from both investigated time points were combined to calculate Shannon indexes for alpha diversity in each nutrition group (ND – green, PA – blue, OA – pink) stratified by age (young–left, light colors; old – right – dark colors) (mean ± SEM). **d** Principal coordinates analysis of beta-diversity based on Bray-Curtis distances shows microbiome diversity in animal samples after 1 and 8 wk DI, grouped by age and nutrition. Shown ND – green, PA – blue, OA – pink; young light, old dark colors. The grey dots represent the data points of the two non-analyzed diets. **e** Relative abundance (%) at phylum level dependent on age and nutrition after 1 and 8 weeks of DI. Data derived from 3 to 7 mice per condition. DI dietary intervention, ND normal diet, PA-ED palmitic acid enriched diet, OA-ED oleic acid enriched diet, *P.g.* Porphyromonas gingivalis infection. Statistical analysis in (**c**): Mann–Whitney U-test, **d**: Permanova statistical test; ***p ≤ 0.001. **a** created with BioRender.

To determine whether nutrition by itself impacts the aged and young murine microbiome (Fig. 2a), young (5 weeks) and old (73 weeks) C57BL5JRj mice received identical diets either supplemented with PA-ED, OA-ED and compared to ND. Fecal samples were analyzed for microbial composition on the taxonomic (Fig. S2a, b) and phylum level (Fig. S2c) one and eight weeks after start of the dietary intervention. Already after one week, PA-ED induced a distinct microbial pattern with increased *Lachnospiraceae* subtypes (e.g., *NK4A136*) and decreased relative abundance (RA) of *Akkermansia* and *Muribaculaceae*. In contrast, conversion to OA-ED supported a microbial composition comparable to respective ND in young and old groups during the first eight experimental weeks (Fig. 2b). Overall, there were only minor age-specific taxonomic differences in OA-ED and ND-fed animals. In contrast, PA-ED rapidly induced changes in MB composition with enhanced alpha diversity reflected in a significant increase in the Shannon index as compared to ND in both age groups (Fig. 2b,c). However, principal coordinates revealed age as a significant factor in beta diversity metrics when comparing respective young versus old dietary groups (ND young vs old: **(*p* ≤0.01); PA-ED young vs old: **(*p* ≤ 0.01); OA-ED young vs old: **(*p* ≤ 0.01)) (Fig. 2d). Furthermore, the microbiome of OA-ED- and ND-fed animals appeared similar and stable in phylogenetic composition, whereas PA-ED stably enhanced the *Firmicutes* to *Bacteroidetes* ratio in both age groups (Fig. 2e).

### Nutritional oleic acid but not palmitic acid stabilizes the gut microbiome and accelerates recovery in response to antibiotic challenge

**Fig. 3:**
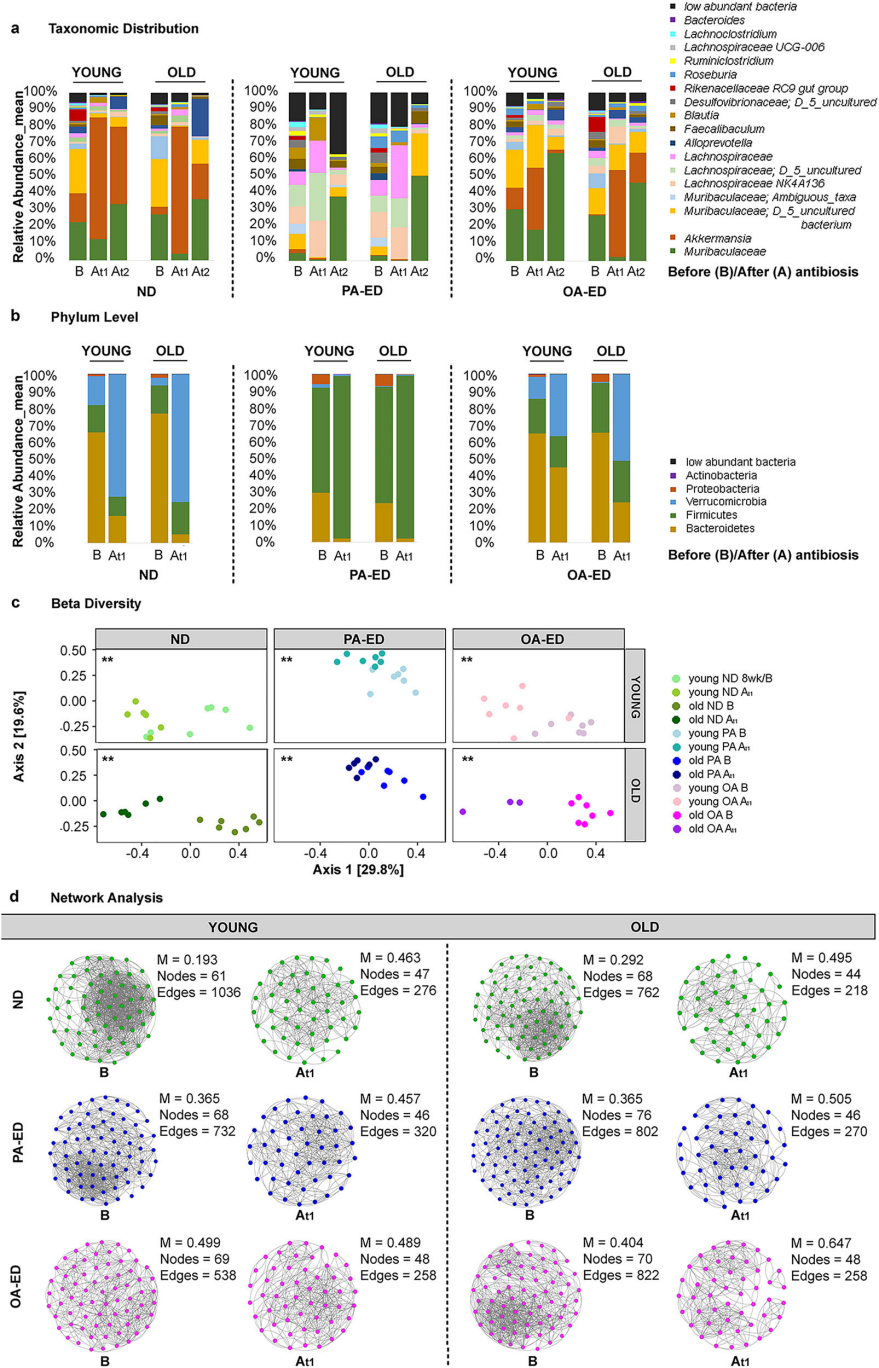
Dietary oleic acid supports recovery of microbiome from antibiosis. **a** Bacterial taxonomic summary of young and old animals receiving dietary intervention with normal (ND), palmitic acid (PA) enriched diet (ED) or oleic acid (OA) ED before (B), 4 days (A_t1_) and 7 weeks (A_t2_) after antibiosis. **b** Relative abundance (%) of bacteria at phylum level dependent on age and nutrition before (B) and after (A_t1_) antibiosis. **c** Principal coordinates analysis of beta-diversity based on Bray-Curtis distances shows microbiome diversity in animal samples before (B) and after (A_t1_) antibiosis, grouped by age and nutrition. **d** Network analyses of microbial composition in different nutrition (ND, PA-ED, OA-ED) and age (young vs old) groups before (B) and after (A_t1_) antibiosis (AB). Besides networks, computed values for modularity (M), nodes and edges are depicted. Data derived from 3 to 7 mice per condition. DI dietary intervention, ND normal diet, PA-ED palmitic acid enriched diet, OA-ED oleic acid enriched diet, AB antibiosis. Statistical analysis in (**c**): Permanova statistical test; **p ≤ 0.01.

To investigate whether dietary intake modulates microbiome resilience in young and old organisms, a five-day antibiotic treatment with oral enrofloxacin was used as a major perturbation. Regardless of diet and age, oral intake of antibiotics fundamentally changed taxonomic distribution at genus (Fig. 3a) and phylum level (Fig. 3b) and beta-diversity-metrics (Fig. 3c). The microbiome of young and old PA-fed animals underwent drastic changes in taxonomic composition (Fig. 3a, B vs A_t1_), in contrast particularly young OA-ED groups exhibited minor changes in their taxonomic microbial composition (Fig. 3a, B vs A_t1_) and on phylum level (Fig. 3b, B vs A_t1_) after antibiotic exposure. Overall, a decrease of *Muribaculaceae* accompanied by a relative increase in *Akkermansia* was detectable in OA-ED and similarly in ND animals in an age-independent manner (Fig. 3a). Regulation of *Akkermansia* is reflected at phylum level in an increase in *Verrucomicrobia* (Fig. 3b). Moreover, the microbiome of PA-ED animals failed to recover from antibiotic-induced changes during the 6 week follow-up (A_t2_), while the microbiome in ND and OA-ED animals returned to compositions similar to its previous state (before antibiosis) during that time period (Fig. 3a; A_t1_ vs A_t2_).

Network analyses and calculated associated modularities (M) revealed that dietary intake of OA-ED improved modularities as compared to PA-ED or ND in both age-groups (Fig. 3d). Of note, OA-enriched nutrition increased considerably modularity in old animals after antibiotic treatment and supported development towards a modularly structured interactive network (M = 0.404 → M = 0.647) (Fig. 3d).

### Oleic acid stabilizes gut microbiome in old periodontally*-*infected animals

**Fig. 4:**
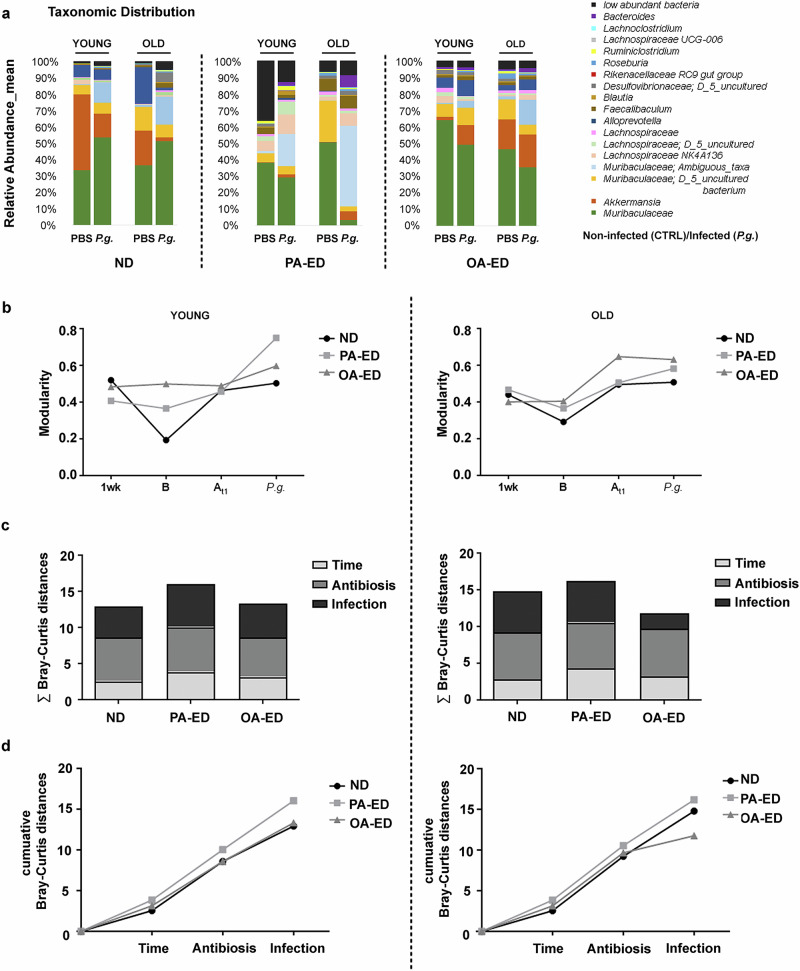
Palmitic acid destabilizes microbiome of old animals making it more susceptible to *P. gingivalis*- induced changes. **a** Relative taxonomic distribution pattern in young and old animals fed either with ND, PA-ED or OA-ED and infected with *Porphyromonas gingivalis* (*P.g*.) in comparison to PBS-infected CTRL animals. **b** Modularity measured at different time points (after 1 week of DI (1wk), after 8 weeks of DI/before antibiosis (8wk/At_1_), after antibiosis, after infection (uninfected also At_2_), young–left, old—right). **c** Sum of Bray-Curtis distances across different treatment phases, including time points (1 wk - 8 wk), antibiotic treatment (beforeAB—afterAB), and infection (afterAB vs. infection with *Pg*). **d** Cumulative Bray-Curtis distance reflects the overall microbiome changes throughout the various treatment phases. (time, antibiosis, infection) (young—left, old—right). Data derived from 3 to 7 mice per condition. DI dietary intervention, ND normal diet, PA-ED palmitic acid enriched diet, OA-ED oleic acid enriched diet, CTRL control, *P.g*. – *Porphyromonas gingivalis*.

We next examined to what extent a subsequent oral inoculation with dysbiosis-inducing periodontal pathogen *P. gingivalis* disturbs the young and old gut microbiome in the presence of the respective nutritional intake. Animals received oral applications of 10^9^ cfu *P. gingivalis* in 2% methylcellulose three times per week for five weeks to induce periodontal disease. To avoid cross-contaminations, samples were taken four days after the last infection. Analysis of microbial colonization revealed no relevant detectable proportion of *P. gingivalis* (Fig. 4a). However, PA-ED/old animals in particular showed differences in microbiome composition in response to *P. gingivalis*-inoculation as compared to their respective control groups, specifically a reduced abundancy of *Muribaculaceae* in periodontally-infected animals (Fig. 4a).

Microbial modularities of OA-ED/young and ND/young animals appeared stable over time and interventions as compared to PA-ED/young animals (Fig. 4b, left). In old animals, the modularities changed in an almost parallel manner from A_t1_ onwards in all groups (Fig. 4b, right); nevertheless, modularities were consistently highest in old OA-ED animals.

The cumulative distances of beta-diversity did not change in response to the different interventions—being time (dietary intake of respective diet), antibiosis and infection - in young OA-ED animals as compared to ND. In contrast, the microbiome of animals that received PA-ED exhibited changes in microbial composition in response to external stimuli as depicted by an increase in cumulative Bray-Curtis distances (Fig. 4c, left). However, in old animals, intake of dietary OA was accompanied by less microbial changes as compared to old PA-ED and ND groups in response to periodontal infection (Fig. 4c, right). Effects of diets were further underlined by the calculation of cumulative distances of beta-diversity (Bray-Curtis distances) (Fig. 4d) showing highest microbiome diversity changes in young animals under PA-ED (Fig. 4d, left), while in old animals, especially in the presence of periodontal infection, intake of OA-ED stabilized microbiome diversity as compared to PA-ED and ND (Fig. 4d, right).

### Substitution of dietary oleic acid enhances serological stress-reducing mediators in periodontally infected young and old mice

**Fig. 5:**
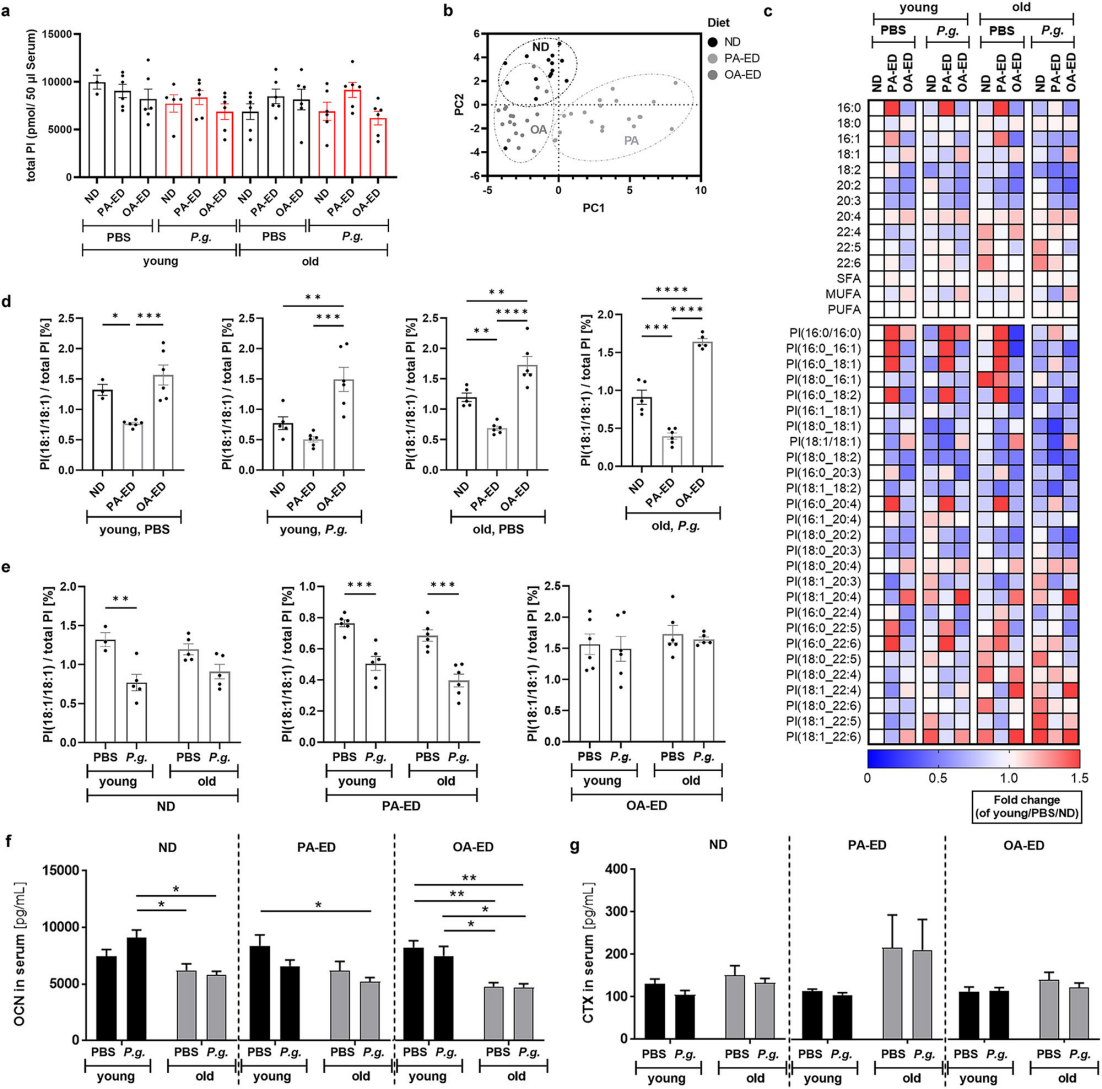
PI(18:1/18:1)-serum levels correlate with quality of trabecular bone after oral *Porphyromonas gingivalis*-infection. Young (5 weeks) and old (≥73 weeks) C57BL6JRj were fed with different diets (normal (ND), palmitic acid (PA) enriched diet (ED) or oleic acid (OA) ED) for 16 weeks, challenged with antibiotics and infected with PBS or *Porphyromonas gingivalis* (*P.g*.) for 5 weeks. **a** Serum phosphatidylinositol (PI-)-species were analyzed by UPLC-MS/MS for total PI (pmol/50 µl serum). **b** Score plots of the Principle Component Analysis (PCA) depicting differences in PI species (as % of total PI) in relation to dietary intake. Mice pooled according to diet, age and infection. Drawn ellipses indicate the distribution of the three dietary groups. **c** Heatmap showing fold change of PI species or subclasses (% of total PI) in relation to diet, age, infection as compared to the control group (ND young/PBS). Impact of (**d**) diet, (**e)** age and infection on proportion of PI(18:1/18:1) (% of total PI). Data in (**a**, **b**, **e**, **h**, **i**) are given as mean ± SEM, *n* = 3–6 mice. ELISA serum analyses **f** bone formation (osteocalcin—OCN) and **g** bone degradation (carboxy-terminal telopeptide of type 1 collagen—CTX); *n* = 5–6 mice. Statistics were analyzed by (**a**) ordinary one-way ANOVA with Dunnett´s or **b**, **d**, **f**, **g** Tukey´s post hoc tests, **e** two-way ANOVA with Tukey´s post hoc tests. *p < 0.05; **p ≤ 0.01; ***p ≤ 0.001; ****p ≤ 0.0001. ND normal diet, PA-ED palmitic acid enriched diet, OA-ED oleic acid enriched diet, CTRL control, *P.g*. – *Porphyromonas gingivalis*. ***n*** = **3–6/group**.

Since microbiome composition, specifically the presence of PI-producing *Bacteroidetes*, indicated changes in systemic and cellular stress management, we next determined whether OA-ED supports resilience and stress response associated with *P. gingivalis*-infection and age. Thereby, a systemwide serum analysis of stress resilience phospholipid indicators was performed. All samples contained similar amounts of total PI (Fig. 5a). Therefore, a principal component analysis (PCA) score plot was used to evaluate distribution of PI species (as % of total PI) within the individual dietary groups with all respective age and infection groups (Fig. 5b). Specifically, PA-ED groups differed in their serological PI composition as compared to OA-ED and ND groups for which drawn ellipses indicate close proximity (Fig. 5b). Further, analysis of single PI species (depicted as heatmap (Fig. 5c) and Volcano Plot (Fig. S3a)) revealed age-associated differences in PI proportions in uninfected OA-ED- and ND/PBS animals, whereas PI-composition in PA-ED/PBS-animals appeared comparable between young and old animals (Fig. 5c; S2a). Overall, nutritional intake was reflected in systemwide lipidomic profiles, with PA-ED increasing levels of 16:0-containing PIs, and OA-ED at least partially enhancing protective 18:1-PI in both age groups (Fig. 5c). *P. gingivalis*-inoculation of PA-ED/old mice reduced serological 16:0-containing species (Fig. 5c) and induced the most pronounced age-dependent changes in lipidomic composition (Volcano plots in Fig. S3a). In contrast, *P. gingivalis*-infected old OA-ED and ND-animals did not present with a marked shift in PI composition as compared to their respective younger groups.

Dietary intake of PA-ED resulted in lower serological levels of the stress-reducing SCD-1-derived lipokine PI(18:1/18:1) as compared to OA-ED and ND in both age groups (Fig. 5d). Periodontal infection lowered PI(18:1/18:1)-levels significantly even further in PA-ED animals of both ages as well as in young ND-fed mice as compared to respective controls. On the contrary, nutritional substitution of OA-ED stabilized PI(18:1/18:1)-levels in the presence of oral *P. gingivalis*-infection in young and old groups (Fig. 5e).

We next determined whether this was reflected in circulating markers of bone metabolism. Bone formation marker osteocalcin (OCN) was reduced in an age-dependent manner in all dietary groups with no detectable impact of *P. gingivalis*-infection at the time of sacrifice one week after the last oral inoculation (Fig. 5f). Additionally, no acute age-, diet- or infection-associated regulation occurred in serum bone degradation marker carboxy-terminal telopeptide of type 1 collagen (CTX); only PA-ED appeared to double CTX levels in aged animals as compared to its respective controls, although non-significantly and unaffected by oral infection (Fig. 5g).

### Dietary intervention impacts cellular lipid content and response to infection in an age-dependent manner

Nutritional intake and periodontal infection modulated serological PI(18/1/18:1)-stress lipokine levels. To determine whether this is reflected in the local bone microenvironment, we next investigated whether bone marrow precursor cells, regulators of inflammatory processes, contribute to local stress regulation in periodontal infection and its systemic dissemination. Cells were isolated from young and old animals after up to 9 weeks of respective dietary substitution to investigate their PI content and associated cellular bone-remodeling pathways.

**Fig. 6:**
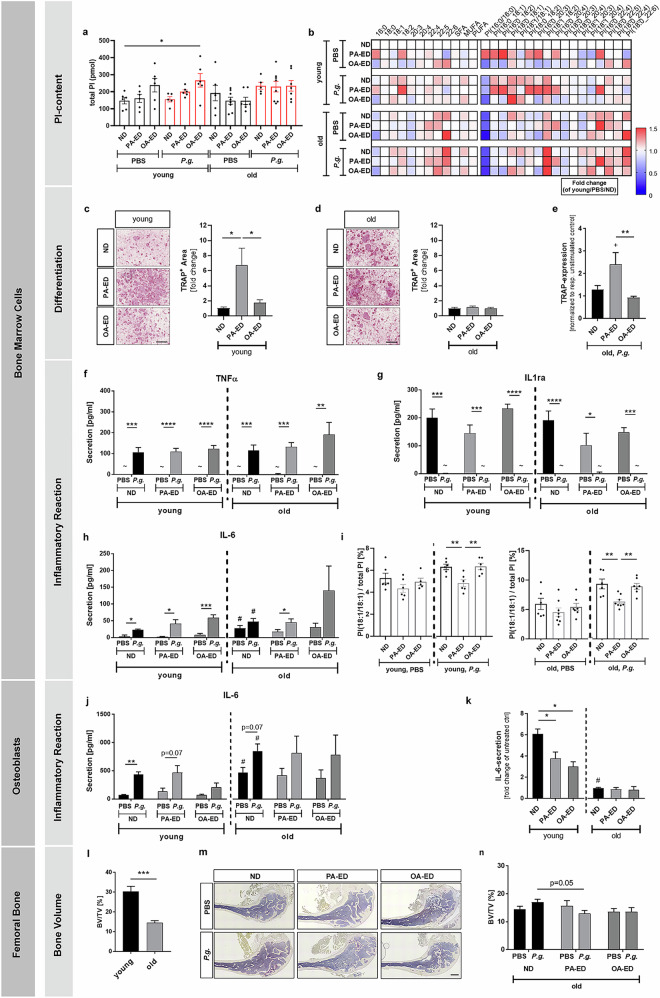
Palmitic acid intake increases inflammation, reduces cellular stress resistance and compromises femoral bone structure in old animals undergoing periodontal infection. Cells were isolated from young (21 weeks) and old (>20 months) mice on dietary intervention PA-ED, OA-ED as compared to ND for up to 9 weeks. **a**, **b**, **i** Cell pellets were analyzed for PI species UPLC-MS/MS. **a** Total PI amount (pmol). **b**, **f–i** 2.5 × 10^6^ bone marrow cells (BMCs) collected after sacrifice, cultured for two days, 6 h infected with PBS or *P. gingivalis*. **b** Heatmaps depicting fold change of untreated control.: PI species proportion and subclasses (% of total PI) according to diet, age, infection as compared to controls (young ND/PBS). TRAP-staining of primed osteoclasts **c** young and **d** old donors. ImageJ-quantification by measurement of osteoclast-covered area- fold change of ND. **e** qPCR-analyses of old differentiated osteoclasts 6 h *Porphyromonas-gingivalis*-infection (relative normalized expression levels (RNE) of respective controls). Cell culture supernatants of primed BMCs were investigated for **f** TNFα-, **g** IL1ra- and **h** IL-6-secretion using ELISA- depicted in pg/ml as mean ± SEM. **i** cell pellets analyzed for diet-, age- and infection-specific proportion of PI(18:1/18:1) (% of total PI). Data as mean ± SEM, *n* = 5–8. IL-6-ELISA from cell culture supernatant of primed osteoblasts from young (21 weeks) and old (>20 months) C57BL6JRj in (**j**) pg/ml and **k** depicted as fold change of respective untreated control (*n* = 3). **l-n** histomorphometric analyses of TRAP-stained femoral slices depicting relation of bone volume (BV) to total volume (TV) (BV/TV). **h** Changes in BV/TV during ageing process in ND-fed animals. **i**, **j** Diet- and infection-dependent changes in femoral bone of old animals. Statistical analysis in ordinary one-way ANOVA **a**, **j** Dunnett´s or **c–e**, **f–k** Tukey’s post hoc tests within treatment groups, two-tailed Student’s *t* test for pairwise comparisons. ~ below detection limit; pairwise comparison to respective unstimulated control ^+^*p* < 0.05 (in **e**); pairwise comparison between young and old ^#^ *p* < 0.05, ^##^ *p* ≤ 0.01 (in **h**, **j**, **k**); *p < 0.05; **p ≤ 0.01; ***p ≤ 0.001; ****p ≤ 0.0001. ND normal diet, PA-ED palmitic acid enriched diet, OA-ED oleic acid enriched diet, CTRL control, *P.g*. *Porphyromonas gingivalis*. Scale in (**m**): 400 µm.

Overall, BMC-PI content was comparable in all dietary groups (Fig. 6a). However, reflecting the systemic serological profiles, the depicted heatmap demonstrates enhanced 16:0-containing PI-species and lowered PI(18:1/18:1)-levels in BMCs derived from young PA-ED-animals (Fig. 6b). *P. gingivalis*-infection reduced PI(16:0/16:0)-levels. In contrast, PA-ED did not raise the proportion of total 16:0-containing PI or individual species in cells of old animals (Fig. 6b).

Next, direct dietary priming of bone marrow cell (BMC)-derived precursor cells was investigated using a five-day incubation with stimulatory factor Receptor Activator of NF-κB Ligand (RANKL) to induce osteoclastic differentiation. Differentiation was significantly enhanced in BMC-cultures derived from young PA-fed animals as compared to cultures derived from young OA- or ND- groups (Fig. 6c), whereas osteoclastic TRAP- staining of BMCs isolated from old animals did not reveal diet-associated differences (Fig. 6d). However, expression of osteoclastic marker *Trap* revealed a significant increase in cells derived from aged PA-groups in response to *P. gingivalis*-infection as compared to respective controls and cells derived from old OA-fed mice (PA/young/P.g. vs PA/old/P.g.: ^+^ *p* < 0.05; PA/old/P.g. vs OA/old/P.g.: **p ≤ 0.01;) (Fig. 6e). Additionally, BMCs were examined for their secretory profile of inflammatory markers in relation to dietary priming through nutritional intake. Three days after cell-isolation from young and old animals, *P. gingivalis*-infection induced a significant, however, diet- and age-independent, increase in pro-inflammatory tumor necrosis factor alpha (TNFα)- and decrease in anti-inflammatory interleukin-1 receptor antagonist (IL1ra)-secretion (Fig. 6f, g). Though, IL-6-secretion appeared differentially regulated in age groups in response to *P. gingivalis*-infection. Whereas in young animals a significant and comparable increase was detectable in all dietary groups, *P. gingivalis*-infection induced a significantly lower secretory increase in BMCs isolated from old animals, which was only significant under PA-ED (Fig. 6h).

Since the cellular inflammatory reaction was uniformly enhanced by the infectious stimulus despite the depicted differences in osteoclastic differentiation, BMC content of stress-reducing PI(18:1/18:1) was investigated for age-defined response to nutritional modulation and bacterial infection. Specifically, BMCs derived from PA-ED animals were susceptible to *P. gingivalis*-induced changes in PI-composition as indicated by volcano plots (Fig. S3b). In contrast to serological data, nutrition by itself did not affect PI(18:1/18:1)-levels. However, *P. gingivalis* infection significantly decreased PI(18:1/18:1)-abundancies in cells derived from young and old PA-ED-animals as compared to OA-ED and ND (Fig. 6i).

### Age and dietary intake impact osteoblastic secretion of inflammatory mediator

To investigate whether bone-forming osteoblasts, counterregulators of BMC-derived osteoclasts and stabilisators of the local bone microenvironment, show long-lasting differences in their inflammatory IL-6 response, osteoblasts isolated from PA-ED, OA-ED and ND groups were cultured for up to three weeks without supplementing additional fatty acids. Basal IL-6-secretion was comparable and unaffected by dietary intake in young and old age groups. *P. gingivalis*-infection significantly increased IL-6 in young ND-derived cells (Fig. 6j). A significant six-fold increase in IL-6 secretion was detected in young ND cells, whereas response of FA-ED-derived cells was significantly lower - depicting only a threefold increased secretion (Fig. 6k). Basal IL-6 secretion of cells derived from old ND-animals appeared enhanced as compared to cultures extracted from young animals (ND/young vs ND/old). However, their responsiveness to *P. gingivalis-*infection was significantly lower (ND/old vs ND/young: ^#^ *p* < 0.05). Overall, cultures did not show significant nutrition-dependent impact, however, manifesting age as defining factor.

### Dietary palmitic acid enhances femoral bone loss in response to *P. gingivalis*-infection in aged animals

Cellular data indicated that the inflammatory response of osteoblasts is primarily regulated in an age-dependent manner, whereas BMCs appeared as differential regulators of age-, nutrition- and infection-driven stress responders in the local bone microenvironment.

In general, femoral bone mass was reduced in untreated aged as compared to young animals on ND (ND/old vs ND/young: ***p ≤ 0.001) (Fig. 6l). However, nutritional intake of PA-ED led to a reduction in BV/TV in old animals challenged with *P. gingivalis* as compared to aged ND/P.g.-animals (ND/old/*P.g*. vs PA-ED/old/*P.g*.: *p* = 0.05) (Fig. 6m, n).

## Discussion

With a prevalence of more than 1 billion cases worldwide, severe PD is one of the most abundant noncommunicable diseases worldwide^43^. Although present in young patients as well^44^, periodontitis is mainly associated with age and described as one of the most prevalent diseases in the elder population, affecting the tooth-supporting tissues^45^ and being connected to a great amount of comorbidities^46^. New understanding of the pathogenesis of periodontitis involves the intricate interplay between inflammation, polymicrobial biofilm formation and associated tissue loss^47^. This model describes periodontitis as multifactorially induced, with the interaction between bacterial restructuring and inflammatory responses determining disease establishment. *P. gingivalis* is considered a periodontal key pathogen in this process for being active in driving dysbiosis and the inflammatory milieu in disease progression. It emphasizes the sensitive balance between inflammation, dysbiosis, and their resolution in shaping the course of periodontitis. Moreover, based on connection to systemic diseases, systemic spread of the orally induced factors could increase disease susceptibility and compromise systemic tissue homeostasis. Previous findings from our group connected oral *P. gingivalis*-infection to loss in femoral bone microarchitecture and demonstrated immunomodulatory potential of dietary fatty acids in alveolar and systemic bone loss in comorbid obese and normal weight young mice^4,6,15^. We now determined age-defined systemic changes in response to oral periodontal infection and whether dietary fatty acids act as sufficient modulators.

Our data demonstrate that PA-ED, unlike OA-ED, exacerbated *P. gingivalis*-associated oral bone loss, particularly in aged animals. Systemically, PA-ED intake fostered destabilization of gut microbiota, increasing susceptibility to disturbances and amplifying infection-driven microbial shifts. Furthermore, PA-ED reduced serological and cellular stress resistance as compared to OA-ED and promoted cellular priming, thereby enhancing osteoclastic differentiation in young and old *P. gingivalis*-challenged bone marrow cells of the bone microenvironment. Osteoblasts exhibited age-related basal inflammation with reduced responsiveness to infectious stimuli, creating a pro-inflammatory microenvironment conducive to PA-driven destruction. This was corroborated by heightened periodontal infection-induced femoral bone loss in PA-ED-fed old mice, indicating age to be sufficient as comorbidity.

Therapeutic modulation of the bacterial-induced inflammatory host response is an emerging focus in addressing sequelae of periodontal disease and increasing clinical evidence suggests that diet - including fat composition - plays a significant role. Specifically, a diet enriched with saturated fats is associated with PD progression in the elderly^42^. Contrary, a cross-sectional study identified a significant association between MD adherence, reflected in plant based foods, fish, olive oil, and lower odds of being affected by periodontal disease^48^, further supported by a recent study demonstrating that low MD adherence increased risk for stage III/IV periodontitis in a university patient cohort^49^. Several MD components are being discussed for their protective potential^50^, thereby, OA, most common unsaturated FA in human nutrition and serum^51,52^, is not only fuel for generating energy but acts immunomodulatory^19,53,54^. Recently, it was demonstrated that OA-serum levels correlated negatively with periodontal pocket depth (PD)^13^ in study participants on an optimized anti-inflammatory diet^12^. In contrast, a positive correlation between levels of saturated FA, specifically palmitic acid (PA (C16:0)), periodontal probing depth and clinical attachment levels had been described^14^. Of note, 6-week- substitution of WD with MD reduced gingival inflammatory parameters despite constant plaque levels^11^, underlining potency of nutritional interventions in fighting infectious stimuli. However, underlying mediators remain to be elucidated. Therefore, we transferred this scientific question to an animal model and investigated nutritional potency on age-associated impact of *P. gingivalis*-induced periodontal disease.

The oral cavity and the gut are physically connected^55^ and it has already been shown that bacterially induced periodontitis affects gut microbiome^22^. Of note, fecal transplantation can restore the oral microbiome and alleviate oral bone loss, indicating a tight interplay between the oral and gut microbiome^56^ and providing an indication for therapeutically targeting a disseminating periodontal infection. Previous findings demonstrated the beneficial effects of the Mediterranean diet on the human gut microbiome^57^. Our data demonstrated improved microbiome stability and resilience towards antibiosis and periodontitis-promoting *P. gingivalis*-infection especially in young OA-ED-fed mice. Although an enhanced intake of mono-unsaturated OA did not induce significant changes in microbiome composition as compared to ND. Nevertheless, the preventive qualities of OA-ED became specifically apparent in individuals challenged by antibiosis and *P. gingivalis* infection since taxonomical and phylum composition were largely stable, as underlined by lowered Bray-Curtis-distances, with high modularity of microbiota and consistent microbiome recovery after antibiosis. In contrast, the microbiome of PA-ED-fed animals appeared to be more susceptible to *P. gingivalis*-induced changes accompanied by enhanced femoral bone loss in old PA-ED-fed animals indicating an increased systemic dissemination of periodontitis in this group. Furthermore, antibiotics could exert long-term effects on microbiome composition and stability^58,59^, which OA-ED protected from by demonstrating rapid recovery as opposed to PA-ED, which could have interfered with host response to subsequent infections and could be the subject of detailed future studies such as *P. gingivalis* infection with and without antibiosis in isolation.

Dietary intake of unsaturated Western diet-derived PA in an otherwise identical diet led to substantial alterations in composition of gut microbiome with an increase in alpha-diversity metrics and changes in beta-diversity. Although it had previously been suggested that an enhanced alpha-diversity could be a sign of gut microbiota health and is associated with disease status^60^, recent work did not detect a correlation between alpha diversity and bone mineral density in elderly people - suggesting additional factors^61^. Furthermore, our study indicates that the number of different taxa might be less important than the species present and the stability of the assemblage. PA may accelerate ageing processes in the murine microbiome as opposed to OA, since previous studies have shown specific upregulations (*Lachnospiraceae NK4A136*)^62^ and downregulations (*Akkermansia*)^63^ during ageing in mice. This is underlined by PA-induced changes of the phylogenetic F/B-ratio and corroborated with previous observations in human^64^ and murine ageing^65^. Moreover, ingestion of PA-ED impaired microbiome recovery after antibiotic treatment not only in old but also young mice, similarly observed in aged mice in a previous study^62^. Systemic antibiotics as a previously very common therapeutic periodontal intervention can affect both the oral and intestinal microbiome accompanied by oral bone loss^56^. Overall, PA-ED-fed animals displayed reduced modularity in their microbiome indicating overall destabilization when faced with external challenges. Thereby, oral infection with *P. gingivalis* induced marked changes age-independently in PA-ED-challenged microbiome underlining its influenceable state.

Intake of PA-ED made the microbiome more susceptible to *P. gingivalis*-induced changes accompanied by reduced stress-resilience. Previously we showed that dietary intake of PA-ED, in contrast to OA-ED, induced enhanced osteoclastic abundancy in femoral bone in response to oral *P. gingivalis*-infection^4^. In line, BV/TV-measurements in old animals depicted disturbed bone microarchitecture in animals on PA-ED with a reduced bone volume in *P. gingivalis*-infected animals. Serological signs of systemic inflammation were not present. However, in vitro data demonstrated enhanced basal inflammation in osteoblasts isolated from old mice indicating presence of a compromised inflammatory microenvironment. Similarly, PA-treatment exacerbated existing inflammation in gingival fibroblasts exposed to *P. gingivalis* lipopolysaccharides and pro-inflammatory TNFα^6^. Therefore, while cell priming may play a secondary role in old age, the ageing process itself leads to increased cellular basal cytokine release. A localized inflammation likely contributes to the overall inflammatory microenvironment, potentially acting as a comorbidity that intensifies bone loss under PA in the presence of periodontal disease.

Massive changes in gut microbial compositions went along with differential diet-specific serological PI-composition. Serological analyses of PA-ED-fed animals revealed increased levels of PI(16:0)-metabolites^4^. This was paralleled by persistent incorporation of 16:0 into PI in primed bone marrow cells from young PA-ED-fed animals, an occurrence not observed in cells isolated from old animals. Consistently, accumulation of cytotoxic DAGs coincided with increased RANKL-mediated osteoclast differentiation^17^. Nevertheless, in line with in vivo data depicting reduced BV/TV in PA-ED/*P.g*./old animals, PA-primed osteoclasts from old animals exhibited increased *Trap*-expression under *P. gingivalis* infection, suggesting an altered bone microenvironment and aligning with the reduction in alveolar and femoral bone architecture in PA-ED animals after *P. gingivalis*-infection. In priming experiments, there were overall minimal effects of dietary priming detectable on the inflammatory secretome of bone marrow cells infected with periodontal pathogen *P. gingivalis*. However, IL-6 secretion was significantly enhanced in primed BMCs derived from old PA-fed animals following infection. In line, there was a persistent reduction in the lipokine PI(18:1/18:1) in PA-primed *P. gingivalis*-infected bone marrow cells; supporting previous findings from infected tibial bone under lipotoxic PA-ED-induced stress^19^ and indicating compromised stress resistance in these infected animals. In contrast, OA-ED groups depicted elevated levels of stress-resilience-mediating PI(18:1/18:1) independent of infection as compared to the other dietary groups. This lipokine is known to be upregulated in cells and tissues with enhanced stress tolerance, associated with increased cellular proliferation and reduced cell death^19^. The heightened serum concentration of PI(18:1/18:1) under OA-ED suggests an enhanced limitation of stress-signaling, potentially exerting a beneficial effect during establishment of periodontal disease. Consistent with this, young osteoblasts isolated from mice following OA dietary intervention exhibited sustained resistance to *P. gingivalis*-stimuli by not showing an increase in inflammatory reaction.

Although diet, age, and *P. gingivalis* infection strongly correlate with PI(18:1/18:1) availability, its precise contribution to stress protection under our experimental conditions and the dominant underlying mechanisms remain unclear, which is a limitation of our study. Based on multiomics analysis and functional compensation studies, we previously proposed that PI(18:1/18:1) suppresses the expression of the catalytic subunit of protein phosphatase 2 A (PPP2Aca), thereby modulating stress-activated protein kinases, activating the master autophagy regulator ULK1, initiating the unfolded protein response, and inactivating the pro-survival extracellular signal-regulated kinase (ERK)1/2^19^. Indeed, infection is associated with altered stress tolerance^66^, largely mediated by *P. gingivalis* through these and related pathways^67–75^, hinting towards a critical role for PI(18:1/18:1) in this process. However, the exact mechanisms remain elusive.

Our study included only male animals to purposely limit the influence of hormonal changes in aged female mice on bone metabolism^30^, which could be addressed in future studies. Additionally, time of day can impact microbiome composition^76^. We, therefore, scheduled sampling times to mitigate this factor. We were unable to detect *P. gingivalis*, indicating that it was not able to manifest itself sufficiently in the intestine. Nevertheless, *P. gingivalis*-associated changes were detectable orally and in gut microbiome composition. While murine and human microbiomes share similarities, significant differences might exist when comparing predominant phyla^77^. However, our analysis focused on specific microbial resilience and relevant changes in species with PI-producing Bacteroidetes^33^ representing a potential connection between stress resistance, cellular and tissue homeostasis, and microbiome composition. Overall, analysis of microbial composition using amplicon sequencing identified biologically relevant differences in microbial communities. Resulting taxonomic shifts are often accompanied by functional changes, as many microbial functions are closely linked to specific taxa that could contribute to the resilience of the microbiome^78^. For example, resilience plays a crucial role in the oral microbiome since a stable microbial balance is essential for maintaining oral health^79^. Thereby, the significant differences in microbial composition in our experimental groups could reflect relevant biological variations that are interpretable even without direct functional analyses. Nevertheless, current important results could be starting point for future metagenomic and metabolomic analyses that support deeper functional insights.

Periodontitis is a complex, bacterially-induced disease^80^ and alternative models using ligatures or bacterial mixtures exist. However, we purposely chose a model involving a single bacterium to align mechanism and compare findings to our previous data^4,6^. It provided us with the opportunity to specifically study the impact of diet and age in understanding the broader implications for oral health and systemic tissue dynamics. Nevertheless, swallowing oral bacteria may have induced systemic changes due to quantity and independent from its inherent virulence. Though, we did not detect *P. gingivalis* in the gut microbiome four days after the final infection, virulence factors, systemic dissemination capability, and pathogenic potential of *P. gingivalis*^7,81,82^ and a direct effect of *P. gingivalis* cannot completely be ruled out. However, the comorbid association of PA-ED with a *P. gingivalis* infection in particular demonstrated an intensification of infection-induced effects such as weakened microbiome, reduced stress resistance and worsened oral and systemic bone quality. Whether this is exclusively carried by the immune system or supported directly by indigested *P. gingivalis*, remains to be elucidated. Overall, OA-ED is protective, stress-reducing and counteracts the damaging impact of *P. gingivalis* - whether direct or indirect.

In summary, our data indicate nutrition as a potent immunomodulatory tool to modify dissemination of periodontal infection in age: Firstly, microbiome stabilization appears to reduce susceptibility to periodontal disease. Secondly, cellular changes, particularly age-dependent alterations in inflammatory potential, exacerbate the detrimental effects of PA-ED and periodontal infection. Conversely, animals on an OA-enriched diet exhibited increased stress tolerance, coupled with a diminished cellular inflammatory response to *P. gingivalis* and improved bone quality. These findings underscore the intricate relationship between dietary fats, gut microbiome stability, cellular inflammatory responses and their collective impact on oral health outcomes.

The long-lasting effect and the simple and economic feasibility of FA supplementation suggest its usage for precision nutrition and individualized approaches in disease control. Thereby, such preventive nutritional non-pharmacological substitution strategies might offer health benefits to address challenges of current demographic developments with increased disease susceptibility and associated economic burden. The here described connection of distant organ systems is another example for the possibility of early detection in personalized medicine that could transform treatment algorithms in the future.

## Material and methods

### Animals

All animal procedures were performed according to the ARRIVE guidelines and the German Law on the Protection of Animals/European Communities Council Directive (86/609/EEC). Animal experiments were approved by the Thuringia State Office for Food Safety and Consumer Protection (UKJ-17/036).

The animals were kept under appropriate barrier conditions in a 14/10-h light/dark cycle. The mice received food and water *ad libitum* and were kept in groups of two to three in individually ventilated cages (IVC) according to their feeding and infection conditions. All cages were located in the same room. To reduce stress, all procedures were carried out simultaneously whenever possible.

### Animal experiments

Animal experiments were performed as described previously^4,6,15^. Briefly, for in vivo investigations, 4 (‘young’) or ≥ 72 (‘old’) weeks old male C57BL6JRj mice (Janvier, CS 4105 Le Genest St Isle, 53941 Saint Berthevin Cedex, France) were randomly divided in different nutritional groups: (normal diet (ND), palmitic acid enriched diet (PA-ED) or oleic acid (OA–ED); 12 animals per group) (Table S1), with PA-ED and OA-ED being isocaloric. After one week of acclimatization, animals were kept on respective diets for up to 9 weeks to investigate subclinical cellular impact and for analyzing morphological changes for 16 weeks based on previous studies^4,6,15^. In line with previously published data^4,6,15^, animals received 0.1 mg/ml enrofloxacin in their drinking water over a period of 4 days after 8 weeks of dietary intervention. After three days without antibiotic treatment, half of the animals were orally infected with 10^9^ cfu *P. gingivalis* in 2% methylcellulose three times per week over a period of 5 weeks. Dose, duration and frequency of *P. gingivalis*-inoculation was performed in accordance to previously published data^4,6,15,83^. The other animals were treated with 2% methylcellulose in phosphate-buffered saline (PBS) as placebo (‘control’).

### Experimental periodontal disease

*P. gingivalis* W50^83^ (American Type Culture Collection (ATCC53978) (Manassas, VA)) was grown in defined medium as described before^84^, and administered via oral gavage at week 10 of FA-enriched-diet feeding. Before bacterial inoculation, animals received 0.1 mg/ml enrofloxacin over their drinking water for four days. For oral infection, *P. gingivalis* was pelleted with a concentration adjusted to 10^9^ cfu in 50 µl of 2% methylcellulose in PBS. Animals were infected three times a week for a total of 5 weeks (n = 6/group). Control animals received 50 µl of 2% methylcellulose in PBS as placebo-infection. Animals were inspected daily, with scorings three times a week paralleling infections and sacrificed one week after final infection.

### Microbiome sampling

Murine fecal samples were taken fresh from individually identified animals in each group as part of the scoring. Based on the known deviations in microbiome composition in the circadian rhythm^76^, sampling always took place at the same time of the day. The fecal pellets were immediately frozen on dry ice and stored at –80 °C until use.

### Extraction from microbiome samples

Genomic DNA was extracted from mouse stool samples using the innuPREP Stool DNA isolation Kit (Analytik Jena, Germany), following the manufacturer’s instructions. The isolated DNA was then used for sequencing library preparation.

### Library Construction and 16S rRNA Gene Amplicon Sequencing

Library preparation and amplicon sequencing were conducted based on methods described by Caporaso and Walters et al.^85,86^. Briefly, the sequencing libraries were prepared using F515-fusion primers with Golay barcodes and R806-constructs (Table S2, supplements). PCR amplification was performed in 50 μl reactions using a CAS-1200 pipetting robot (Qiagen) and a Thermal Cycler S1000 (BioRad, Germany) with Platinum PCR SuperMix (Thermo Fisher Scientific, Germany). The thermal cycling conditions included an initial denaturation at 94 °C for 3 min, followed by 35 cycles of 94 °C for 15 s, 58 °C for 20 s and 72 °C for 30 s, with a final extension step at 72 °C for 10 min. PCR products were quantified using a TapeStation 2200 (Agilent Technologies) with D1000 Tapes, equimolarly pooled and size-selected on 2% SizeSelect E-Gels (Thermo Fisher Scientific, Germany). Sequencing libraries were then prepared for Illumina sequencing using the MiSeq Reagent Kit v2 (Illumina) according to the manufacturer’s protocol. The sequencing run was conducted on an Illumina MiSeq system with 251 cycles.

### Sequencing data analysis

Raw reads were demultiplexed using the QIIME2 bioinformatics platform^87^. Sequence quality and subsequent data processing were performed using the q2-dada2 plugin. This module include sequence denoising, chimera removal, singleton filtering, merging of paired-end reads and dereplication^88^. Taxonomic classification was performed with a pre-trained Naive Bayes classifier on the SILVA REF NR 99 database (release 132) using the 515 F (GTGYCAGCMGCCGCGGTAA) and 806 R (GGACTACNVGGGTWTCTAAT) primers targeting the V4 region of the bacterial 16S rRNA gene^89^. The resulting amplicon sequence variants (ASVs) were utilized for further calculation of microbiome diversity metrics.

### Network analyses

To evaluate non-random bacterial interactions, the SparCC algorithm^90^, implemented in the R package SpiecEasi v.1.1.2, was used for co-occurrence network analyses using the generated feature table. Pairwise median correlations were computed with twenty iterations and the statistical significance of each correlation was determined by bootstrapping with 100 iterations. Significant (*p* < 0.05) SparCC correlations were incorporated into the network analysis. Each feature was represented as a node, with significant correlations depicted as edges. Additional measures of network stability, including modularity indices, were used for further analysis^91^. Networks were visualized using the Gephi platform^92^.

### Histomorphometric analysis of alveolar bone

Processed mandibular bones were fixed in 4% paraformaldehyde (PFA) overnight at 7 °C. The following day, bones were washed twice in PBS and stored in PBS at 7 °C until further usage. Following fixation, lower jawbones were decalcified in 25% EDTA for a total of two weeks with the solution being changed three times per week that was interrupted by one hour soaking in distilled water. The jaws were drained in an ascending row of alcohol, embedded in paraffin and cut in 4 µm thick slices. Slices were stained for tartrate-resistant acid phosphatase (TRAP) and counterstained with hematoxylin to identify TRAP^+^ osteoclasts. Microscopic pictures were taken using a Jenaval lightmicroscope (Carl Zeiss Jena) equipped with an AxioCam MRc5. Pictures were taken using a 12.5–fold objective and single pictures were combined using Image Composite Editor Software (Microsoft). The distance between the cementoenamel junction (CEJ) and the upper edge of the alveolar bone crest (ABC) was measured in the proximal bone peak between the first and second molars and normalized to ND/PBS of respective age group^93^.

TRAP^+^ osteoclasts were counted at the interproximal bone lining the distal aspect of the first molar as described before^94^. In addition, lacunar areas were determined in the distal area of M2 (mm)^94^. Histomorphometric data were normalized to uninfected ND-controls of respective age group and depicted as fold change. Each data set contained up to six individuals based on previous experiments.

### Histomorphometric analysis of femoral bone

Manually defleshed femurs were immersion fixed in 4% PFA overnight. Bones were decalcified in 25% EDTA for a total of two weeks. Paraffin slices of 4 µm were stained for TRAP and counterstained with hematoxylin. Microscopic pictures were taken using an Axio Vert.A1 (Zeiss) using Zen-software. Trabecular bone volume was measured in epiphysis and below the growth cone using ImageJ and normalized to whole tissue area as BV/TV. Histomorphometric data were normalized to uninfected ND-controls and shown as fold change. Each data set contained of six individuals.

### Osteoclast and osteoblast cultures

Osteoclasts and osteoblasts were isolated from bone marrow cells and long bones of young (21 weeks) or old (>20 months) animals after up to 9 weeks of dietary intervention.

### Osteoblasts

Primary osteoblasts were isolated from long bones of fore and hind limbs. After removal of bone marrow, bones were washed in PBS and cut in small pieces prior to collagenase digestion (500 U/ml Collagenase II (Worthington Biochemical Corporation, New Jersey, USA) in DMEM) for 2 h at 37 °C. Afterwards, bone fragments were washed three times in proliferation medium (DMEM low glucose (Thermo-Fisher Scientific)), 100 U/ml penicillin, 100 µg/ml streptomycin (Thermo-Fisher Scientific), 50 µg/ml Gentamicin (Sigma-Aldrich), 1.25 µg/ml Amphothericin B (Sigma-Aldrich) and transferred to a T25-flask with 5 ml proliferation medium. Medium was changed for the first time after 5 days of incubation. Subsequent media changes were performed three times a week until cells reached confluency. For infection and differentiation experiments, cells were seeded in a density of 2.8 × 10^4^ cells per well on 24-well-plates. After two days in culture, proliferation medium was exchanged for differentiation medium (proliferation medium supplemented with 100 µg/ml L-ascorbic acid, 10 mM β-glycerol phosphate and 100 nM dexamethasone (Sigma-Aldrich)). Prior to *P. gingivalis*-infection, osteoblasts were washed twice with prewarmed D-PBS (w/o calcium, magnesium, Thermo-Fisher Scientific) and covered with 1 ml antibiotics-free culture medium (OB—DMEM low glucose with pyruvate supplemented with 10% FBS (Thermo-Fisher Scientific)). Cells were infected with a multiplicity of infection (moi) of 100 cfu of P. gingivalis in 25 µl PBS. Control cells were supplemented with 25 µl PBS. Cells were incubated for 6 h as described before^15^. Supernatants were collected, centrifuged and stored at –20 °C until further ELISA analysis.

### Bone marrow cells

As described previously^95^, bone marrow cells (BMC) were centrifuged out of long bones of front and hind limbs (10,000 x *g* for 20 s). BMCs were resuspended in proliferation medium (αMEM (Sigma) containing 10% FBS (Gibco) and 1% penicillin/streptomycin) and incubated overnight in 10 ml proliferation medium on a 10 cm dish (per one animal). The following day, non-adherent cells were seeded either for infection or for differentiation.

### Osteoclasts

Bone marrow cells were seeded in a density of 3 × 10^5^ cells per well in differentiation medium (proliferation medium supplemented with 50 ng/ml MCSF and 20 ng/ml RANKL (PeproTech)) on a 24-well-plate. Cells were differentiated up to five days at 37 °C, 5% CO_2_ and 95% humidity. Every two to three days, 80% of media were exchanged. When cells were fully differentiated, they were fixed in 4% PFA prior to TRAP staining or prepared for infection. Infection with *P. gingivalis* was performed after 5 days of differentiation. Cells were supplemented with antibiotics-free αMEM and *P. gingivalis* was applied in a moi of 100 for 6 h. Cells were harvested in TRIzol and analyzed for gene expression.

### Infection of bone marrow cells and osteoclasts

In total, 2.5 × 10^6^ non-adherent BMCs were seeded on 6-well plates in antibiotic-free proliferation medium (αMEM (Sigma) containing 10% FBS (Gibco)). After two days, cells were infected with *P. gingivalis* in a moi of 100. After 6 h, cells were rinsed from the bottom of the plate. Cells and supernatant were separated by centrifugation (5’ 1000 x g). The supernatant was stored frozen until ELISA examinations. The cell pellet was washed once in PBS and kept frozen. Methanol was added to the cells before further treatment.

### Gene expression analysis in cells

RNA was isolated with TRIzol Reagent (Thermo Fisher Scientific, Carlsbad, CA, USA)/1-bromo-3-chloropropane. Purification was performed using RNA Clean and Concentrator-5 kit (Zymo Research, Freiburg, Germany) according to the manufacturer’s instructions. Concentration and purity of RNA was measured with Nanodrop 2000 (Avantor, Radnor, PA, USA). RNA was transcribed to cDNA with SuperScript IV Reverse Transcriptase (Thermo Fisher Scientific, Carlsbad, CA, USA) according to the manufacturer’s protocol. For quantitative RT-PCR gene expression levels Luminaris HiGreen Master Mix (Thermo Fisher Scientific) was used and monitored by qTOWER3 (Analytic JENA). *Gapdh* (glyceraldehyde-3-phospate dehydrogenase) (forward: 5ʹ-TGTGAACGGATTTGGCCGTA-3ʹ, reverse: 5ʹ- ACTGTGCCGTTGAATTTGCC-3ʹ) and *Rps29* (40S ribosomal protein S29) (forward: 5ʹ-GAAGTTCGGCCAGGGTTCC-3ʹ, reverse: 5ʹ-GAAGCCTATGTCCTTCGCGT-3ʹ) served as respective reference genes for *Trap*-expression (Tartrate-resistant-acid-phosphatase) (forward: 5ʹ-CCAATGCCAAAGAGATCGCC-3ʹ, reverse: 5ʹ-TCTGTGCAGAGACGTTGCCAAG-3ʹ). Each primer pair was tested with template dilution series for calculation of efficiency followed by analyses of melting curve and agarose gel electrophoresis to exclude primer dimers. Each condition was analyzed at least in biological triplicate, with technical duplicates per sample.

### TRAP-staining

Osteoclast differentiation was evaluated using TRAP-staining. For this purpose, osteoclasts were pre-differentiated and incubated with conditioned osteoblastic supernatant. Afterwards, cells were fixed with 4% PFA for 10 min, incubated with equal volumes of acetone and ethanol for 1 min and stained for TRAP as described before^15^. Cells remained in ddH_2_O until microscopic photographs were taken using Primovert (Zeiss) and EOS 77D (Canon). Number and size of TRAP^+^ cells were evaluated with ImageJ. Each experiment was normalized to its own control (BSA-treatment). Three independent experiments were performed and biological replicates were seeded in triplicates or more.

### ELISA

Protein detection in serum and cell culture supernatant was performed using ELISA Kits (Mouse Osteocalcin (OCN) and Mouse C Terminal Telopeptide of Type I Collagen (CTX) ELISA Kit purchased from My Biosource, Mouse IL-6 Quantikine ELISA Kit and Mouse TNF-alpha Quantikine ELISA Kit from R&D Systems) according to manufacturer’s protocol. Protein levels were calculated by comparison to a standard curve at an absorbance of 450 nm (Infinite M Nano plate reader, Tecan Life Science). For serological analyses, samples from 5 to 6 individuals were measured depending on availability of material. For cell culture experiments, at least three independent experiments were performed and each sample measured in duplicates.

### Extraction and analysis of phosphatidylinositols

Phospholipids were extracted from cell pellets or serum by successive addition of PBS pH 7.4, methanol, chloroform and saline to a final ratio of 14:34:35:17^19,96^. The organic layer was evaporated using an Eppendorf Concentrator Plus (Eppendorf, Hamburg, Germany) in volatile high vapor pressure mode (V-HV) at 30 °C. The lipid film was subsequently dissolved in methanol and an aliquot was subjected to quantitative analysis by ultra-performance liquid chromatography-tandem mass spectrometry (UPLC-MS/MS). 1-Pentadecanoyl-2-oleoyl(d7)-*sn*-glycero-3-phosphoinositol (ammonium salt) (PI(15:0/18:1-d7: Sigma-Aldrich)), stored at –80 °C in the dark under argon, was used as internal standard.

Chromatographic separation was performed at a flow rate of 0.75 ml/min on an Acquity UPLC BEH C8 column (1.7 µm, 2.1 × 100 mm, Waters, Milford, MA) using an ExionLC AD UHPLC (Sciex, Framingham, MA) with acetonitrile/water, 95/5, 2 mM ammonium acetate as mobile phase A, and water/acetonitrile, 90/10, 2 mM ammonium acetate as mobile phase B. The gradient started at 75% mobile phase A and increased to 85% in 5 min, followed by another linear increase to 100% mobile phase A within 2 min and isocratic elution for 2 min. The column temperature was kept at 45 °C.

Eluted lipids were detected by multiple reaction monitoring using a QTRAP 6500+ Mass spectrometer (Sciex, Framingham, MA) equipped with an electrospray ionization source and operated by Analyst 1.7^19,97,98^. Fatty acid anions of PI were detected in the negative ion mode by multiple reaction monitoring. The curtain gas was set to 40 psi, the collision gas to medium, the ion spray voltage to –4500 V in negative mode, the heated capillary temperature to 500 °C, the sheath gas pressure to 55 psi and the auxiliary gas pressure to 75 psi. The declustering potential was set to -11 V, the entrance potential to –10 V, the collision energy to –46 eV and the collision cell exit potential to –6.2 V. The average signal of the sum of both transitions from [M-H]^-^ to fatty acid anions was used for quantitation.

Absolute amounts of lipids refer to the internal deuterated PI standard and are normalized to the volume of serum. Relative proportions of individual lipid species are given as percentage of the sum of all species detected in the respective subclass (= 100%). To calculate the fatty acyl composition or the fractions of polyunsaturated fatty acids (PUFA) (≥2 double bonds), monounsaturated fatty acids (MUFA) (1 double bond), and saturated fatty acids (SFA) (0 double bond) in PI, the respective species within the subfractions were summed up and are given as percentage of the sum of all species detected. Depending on availability and integrity of material, 3–6 different serological and 5–8 cellular samples were measured.

### Power calculations/data analysis

Based on previous publications^15,16^, a sample size of less than 6 animals in each group was calculated to have a statistical power of more than 80% to detect a difference in bone loss with a significance level of 0.05 (two-tailed, STAT MAT 2.0). The primary objective was to quantify changes in microbial composition and study the presence of specific lipids and periodontal bone loss. One animal was lost during the one week acclimatization period and two additional animals were excluded due to their health status resulting in a final sample size of at least 5 animals per group, which was still in accordance with the power calculation. If not stated differently, 5–6 animals per group were analyzed.

### Statistical analysis

Shannon indices for alpha diversity were compared using the Mann-Whitney U-test. Beta-diversity metrics were calculated to identify significant group clustering patterns of microbiome diversities and the Permanova statistical test was applied. All graphical presentations were generated using R (version 4.3.0) and GraphPad Prism 9 (GraphPad Software v. 9.4.1, USA). A significance threshold of *p* < 0.05 was applied to all statistical tests. Data are expressed as mean ± s.e.m. of *n* independent experiments. Samples were not analyzed in a blinded mode. Data were statistically evaluated by one-way or two-way ANOVAs with Dunnet’s or Tukey’s post hoc tests, or multiple Student’s *t* tests using an α level of 0.05. *P* values of <0.05 were considered statistically significant. Outliers were identified and excluded using a Grubb’s test. Data were also analyzed using Microsoft Excel (Microsoft Office LTSC Professional Plus 2021, Microsoft) and statistical calculations and principal component analysis were performed using GraphPad Prism (GraphPad Software). Heatmaps were created using GraphPad Prism 9.5.1 (GraphPad Software) from relative or absolute intensities that were normalized to control.

## Supplementary information

Revised Supplements

## Data Availability

Raw data from this study are accessible in the SRA database under the Bioproject accession number: PRJNA1132147.

## References

[1] Kennedy, B. K. et al. Geroscience: Linking aging to chronic disease. *Cell* **159**, 709–713 (2014). 25417146 10.1016/j.cell.2014.10.039 PMC4852871

[2] Dugan, B., Conway, J. & Duggal, N. A. Inflammaging as a target for healthy ageing. *Age and Ageing* **52**, afac328 (2023). 36735849 10.1093/ageing/afac328

[3] Santoro, A., Bientinesi, E. & Monti, D. Immunosenescence and inflammaging in the aging process: age-related diseases or longevity?. *Ageing Res Rev.* **71**, 101422 (2021). 34391943 10.1016/j.arr.2021.101422

[4] Doding, A. et al. Immunometabolic capacities of nutritional fatty acids in regulation of inflammatory bone cell interaction and systemic impact of periodontal infection. *Front Immunol.* **14**, 1213026 (2023). 37736098 10.3389/fimmu.2023.1213026 PMC10509849

[5] World Health Organization. Oral health https://www.who.int/news-room/fact-sheets/detail/oral-health: WHO; [updated 14 March 202314 March 2023]. Available from: https://www.who.int/news-room/fact-sheets/detail/oral-health. (2023).

[6] Doding, A. et al. Mediterranean diet component oleic acid increases protective lipid mediators and improves trabecular bone in a Porphyromonas gingivalis inoculation model. *J. Clin. Periodontol.* **50**, 380–395 (2023). 36384158 10.1111/jcpe.13751

[7] Gasmi Benahmed, A., Kumar Mujawdiya, P., Noor, S. & Gasmi, A. Porphyromonas gingivalis in the development of periodontitis: Impact on dysbiosis and inflammation. *Arch. Razi Inst.* **77**, 1539–1551 (2022). 37123122 10.22092/ARI.2021.356596.1875 PMC10133641

[8] Martínez-García, M. & Hernández-Lemus, E. Periodontal inflammation and systemic diseases: An overview. *Front Physiol.* **12**, 709438 (2021). 34776994 10.3389/fphys.2021.709438 PMC8578868

[9] Hajishengallis, G. & Chavakis, T. Local and systemic mechanisms linking periodontal disease and inflammatory comorbidities. *Nat. Rev. Immunol.* **21**, 426–440 (2021). 33510490 10.1038/s41577-020-00488-6 PMC7841384

[10] Schulze-Späte, U. et al. Crosstalk between periodontitis and cardiovascular risk. *Front Immunol.* **15**, 1469077 (2024). 39717783 10.3389/fimmu.2024.1469077 PMC11663742

[11] Bartha, V. et al. Effect of the Mediterranean diet on gingivitis: A randomized controlled trial. *J. Clin. Periodontol.* **49**, 111–122 (2022). 34818686 10.1111/jcpe.13576

[12] Woelber, J. P. et al. The influence of an anti-inflammatory diet on gingivitis. A randomized controlled trial. *J. Clin. Periodontol.* **46**, 481–490 (2019). 30941800 10.1111/jcpe.13094

[13] Kruse, A. B. et al. An exploratory study on the role of serum fatty acids in the short-term dietary therapy of gingivitis. *Sci. Rep.* **12**, 4022 (2022). 35256737 10.1038/s41598-022-07989-5 PMC8901712

[14] Ramirez-Tortosa, M. C. et al. Periodontitis is associated with altered plasma fatty acids and cardiovascular risk markers. *Nutr. Metab. Cardiovasc Dis.* **20**, 133–139 (2010). 19500957 10.1016/j.numecd.2009.03.003

[15] Muluke, M. et al. Diet-induced obesity and its differential impact on periodontal bone loss. *J. Dent. Res.* **95**, 223–229 (2016). 26450512 10.1177/0022034515609882 PMC4720954

[16] Alsahli, A. et al. Palmitic acid reduces circulating bone formation markers in obese animals and impairs osteoblast activity via C16-ceramide accumulation. *Calcif. Tissue Int.* **98**, 511–519 (2016). 26758875 10.1007/s00223-015-0097-z

[17] Drosatos-Tampakaki, Z. et al. Palmitic acid and DGAT1 deficiency enhance osteoclastogenesis, while oleic acid-induced triglyceride formation prevents it. *J. Bone Min. Res.* **29**, 1183–1195 (2014). 10.1002/jbmr.2150 PMC4945760 24272998

[18] Müller, A. K. Olive oil extracts and oleic acid attenuate the LPS-induced inflammatory response in murine RAW264.7 macrophages but induce the release of prostaglandin E2. *Nutrients* **13**, 4437 (2021). 34959989 10.3390/nu13124437 PMC8703532

[19] Thürmer, M. et al. PI(18:1/18:1) is a SCD1-derived lipokine that limits stress signaling. *Nat. Commun.* **13**, 2982 (2022). 35624087 10.1038/s41467-022-30374-9 PMC9142606

[20] Baima, G. et al. Effect of periodontitis and periodontal therapy on oral and gut microbiota. *J. Dent. Res.* **103**, 359–368 (2024). 38362600 10.1177/00220345231222800

[21] Gan, G. et al. Unveiling the oral-gut connection: Chronic apical periodontitis accelerates atherosclerosis via gut microbiota dysbiosis and altered metabolites in apoE(-/-) Mice on a high-fat diet. *Int. J. Oral. Sci.* **16**, 39 (2024). 38740741 10.1038/s41368-024-00301-3 PMC11091127

[22] Kawamoto, D. et al. Oral dysbiosis in severe forms of periodontitis is associated with gut dysbiosis and correlated with salivary inflammatory mediators: A preliminary study. *Front Oral. Health* **2**, 722495 (2021). 35048045 10.3389/froh.2021.722495 PMC8757873

[23] Mann, E. R., Lam, Y. K. & Uhlig, H. H. Short-chain fatty acids: Linking diet, the microbiome and immunity. *Nat. Rev. Immunol* **24**, 577–595 (2024). 38565643 10.1038/s41577-024-01014-8

[24] Xu, A. A. Dietary fatty acid intake and the colonic gut microbiota in humans. *Nutrients* **14**, 2722 (2022). 35807903 10.3390/nu14132722 PMC9268849

[25] Zhang, P. Influence of foods and nutrition on the gut microbiome and implications for intestinal health. *Int. J. Mol. Sci* **23**, 9588 (2022). 36076980 10.3390/ijms23179588 PMC9455721

[26] Duffuler, P., Bhullar, K. & Wu, J. Targeting gut microbiota in osteoporosis: Impact of the microbial based functional food ingredients. *Food Sci. Hum. Wellness* **13**, 1–29 (2023).

[27] Hills, R. D. Gut microbiome: Profound implications for diet and disease. *Nutrients* **11**, 1613 (2019). 31315227 10.3390/nu11071613 PMC6682904

[28] Magne, F. The firmicutes/bacteroidetes ratio: A relevant marker of gut dysbiosis in obese patients?. *Nutrients* **12**, 1474 (2020). 32438689 10.3390/nu12051474 PMC7285218

[29] Okoro, P. C. et al. A two-cohort study on the association between the gut microbiota and bone density, microarchitecture, and strength. *Front Endocrinol. (Lausanne)* **14**, 1237727 (2023). 37810879 10.3389/fendo.2023.1237727 PMC10551180

[30] Pacifici, R. Bone remodeling and the microbiome. *Cold Spring Harb Perspect Med* **8**, a031203 (2018). 28847904 10.1101/cshperspect.a031203 PMC5880157

[31] Seely, K. D., Kotelko, C. A., Douglas, H., Bealer, B. & Brooks, A. E. The human gut microbiota: A key mediator of osteoporosis and osteogenesis. *Int. J. Mol. Sci* **22**, 9452 (2021). 34502371 10.3390/ijms22179452 PMC8431678

[32] Woo, V. & Alenghat, T. Epigenetic regulation by gut microbiota. *Gut Microbes* **14**, 2022407 (2022). 35000562 10.1080/19490976.2021.2022407 PMC8744890

[33] Heaver, S. L. et al. Characterization of inositol lipid metabolism in gut-associated Bacteroidetes. *Nat. Microbiol* **7**, 986–1000 (2022). 35725777 10.1038/s41564-022-01152-6 PMC9246714

[34] Brown, E. M., Clardy, J. & Xavier, R. J. Gut microbiome lipid metabolism and its impact on host physiology. *Cell Host Microbe.* **31**, 173–186 (2023). 36758518 10.1016/j.chom.2023.01.009 PMC10124142

[35] Kindt, A. et al. The gut microbiota promotes hepatic fatty acid desaturation and elongation in mice. *Nat. Commun.* **9**, 3760 (2018). 30218046 10.1038/s41467-018-05767-4 PMC6138742

[36] Sun, Q. et al. SCD1 is the critical signaling hub to mediate metabolic diseases: Mechanism and the development of its inhibitors. *Biomed. Pharmacother.* **170**, 115586 (2024). 38042113 10.1016/j.biopha.2023.115586

[37] Chen, Y. et al. Gut microbiota and bone diseases: A growing partnership. *Front. Microbiol.* **13** (2022). 10.3389/fmicb.2022.877776 PMC9121014 35602023

[38] Bosco, N. & Noti, M. The aging gut microbiome and its impact on host immunity. *Genes Immun.* **22**, 289–303 (2021). 33875817 10.1038/s41435-021-00126-8 PMC8054695

[39] Maynard, C. & Weinkove, D. The gut microbiota and ageing. *Subcell. Biochem* **90**, 351–371 (2018). 30779015 10.1007/978-981-13-2835-0_12

[40] Meng, C., Feng, S., Hao, Z., Dong, C. & Liu, H. Changes in gut microbiota composition with age and correlations with gut inflammation in rats. *PLoS One* **17**, e0265430 (2022). 35290412 10.1371/journal.pone.0265430 PMC8923432

[41] Wu, C. S. et al. Age-dependent remodeling of gut microbiome and host serum metabolome in mice. *Aging (Albany NY)* **13**, 6330–6345 (2021). 33612480 10.18632/aging.202525 PMC7993679

[42] Iwasaki, M. et al. Relationship between saturated fatty acids and periodontal disease. *J. Dent. Res* **90**, 861–867 (2011). 21505204 10.1177/0022034511405384

[43] Geneva World Health Organization. Global oral health status report: towards universal health coverage for oral health by 2023. (2022).

[44] Modin, C. et al. Periodontitis in young individuals: Important factors for disease progression. *J. Clin. Periodontol.* **51**, 74–85 (2024). 37803906 10.1111/jcpe.13884

[45] Hajishengallis, G. Periodontitis: From microbial immune subversion to systemic inflammation. *Nat. Rev. Immunol.* **15**, 30–44 (2015). 25534621 10.1038/nri3785 PMC4276050

[46] Holmstrup, P. et al. Comorbidity of periodontal disease: Two sides of the same coin? An introduction for the clinician. *J. Oral. Microbiol* **9**, 1332710 (2017). 28748036 10.1080/20002297.2017.1332710 PMC5508374

[47] Van Dyke, T. E., Bartold, P. M. & Reynolds, E. C. The nexus between periodontal inflammation and dysbiosis. *Front Immunol.* **11**, 511 (2020). 32296429 10.3389/fimmu.2020.00511 PMC7136396

[48] Altun, E. et al. Association between dietary pattern and periodontitis-A cross-sectional study. *Nutrients* **13** (2021) 10.3390/nu13114167 PMC8621734 34836422

[49] Marruganti, C. et al. Adherence to Mediterranean diet, physical activity level, and severity of periodontitis: Results from a university-based cross-sectional study. *J. Periodontol.* **93**, 1218–1232 (2022). 35119695 10.1002/JPER.21-0643 PMC9544461

[50] Tsigalou, C. et al. Mediterranean diet as a tool to combat inflammation and chronic diseases. An overview. *Biomedicines* **8**. (2020) 10.3390/biomedicines8070201 PMC7400632 32650619

[51] Baylin, A., Kabagambe, E. K., Siles, X. & Campos, H. Adipose tissue biomarkers of fatty acid intake. *Am. J. Clin. Nutr.* **76**, 750–757 (2002). 12324287 10.1093/ajcn/76.4.750

[52] Hernandez, M. L., Sicardo, M. D., Belaj, A. & Martinez-Rivas, J. M. The oleic/linoleic acid ratio in olive (Olea europaea L.) fruit mesocarp is mainly controlled by OeFAD2-2 and OeFAD2-5 genes together with the different specificity of extraplastidial acyltransferase enzymes. *Front Plant Sci.* **12**, 653997 (2021). 33763103 10.3389/fpls.2021.653997 PMC7982730

[53] Fritsche, K. L. The science of fatty acids and inflammation. *Adv. Nutr.* **6**, 293S–301S (2015). 25979502 10.3945/an.114.006940 PMC4424767

[54] Vassiliou, E. K. et al. Oleic acid and peanut oil high in oleic acid reverse the inhibitory effect of insulin production of the inflammatory cytokine TNF-alpha both in vitro and in vivo systems. *Lipids Health Dis.* **8**, 25 (2009). 19558671 10.1186/1476-511X-8-25 PMC2706835

[55] Kunath, B. J., De Rudder, C., Laczny, C. C., Letellier, E. & Wilmes, P. The oral-gut microbiome axis in health and disease. *Nat. Rev. Microbiol.* **22**, 791–805 (2024). 39039286 10.1038/s41579-024-01075-5

[56] Yuan, X. et al. Systemic antibiotics increase microbiota pathogenicity and oral bone loss. *Int. J. Oral. Sci.* **15**, 4 (2023). 36631439 10.1038/s41368-022-00212-1 PMC9834248

[57] Barber, T. M., Kabisch, S., Pfeiffer, A. F. H. & Weickert, M. O. The effects of the Mediterranean diet on health and gut microbiota. *Nutrients* **15**, 2150 (2023). 37432307 10.3390/nu15092150 PMC10180651

[58] Cetinbas, M., Thai, J., Filatava, E., Gregory, K. E. & Sadreyev, R. I. Long-term dysbiosis and fluctuations of gut microbiome in antibiotic treated preterm infants. *iScience* **26**, 107995 (2023). 37829203 10.1016/j.isci.2023.107995 PMC10565780

[59] Patangia, D. V., Anthony Ryan, C., Dempsey, E., Paul Ross, R. & Stanton, C. Impact of antibiotics on the human microbiome and consequences for host health. *Microbiologyopen* **11**, e1260 (2022). 35212478 10.1002/mbo3.1260 PMC8756738

[60] Li, Z. et al. Differences in alpha diversity of gut microbiota in neurological diseases. *Front Neurosci.* **16**, 879318 (2022). 35837118 10.3389/fnins.2022.879318 PMC9274120

[61] Das, M. et al. Gut microbiota alterations associated with reduced bone mineral density in older adults. *Rheumatol. (Oxf.)* **58**, 2295–2304 (2019). 10.1093/rheumatology/kez302 PMC6880854 31378815

[62] Laubitz D. et al. Dynamics of gut microbiota recovery after antibiotic exposure in young and old mice (a pilot study). *Microorganisms* **9** (2021) 10.3390/microorganisms9030647 PMC8003781 33804656

[63] Giri, S. et al. The effect of Porphyromonas gingivalis on the gut microbiome of mice in relation to aging. *J. Periodontal Res.* **57**, 1256–1266 (2022). 36251393 10.1111/jre.13062

[64] Vaiserman, A. et al. Differences in the gut Firmicutes to Bacteroidetes ratio across age groups in healthy Ukrainian population. *BMC Microbiol* **20**, 221 (2020). 32698765 10.1186/s12866-020-01903-7 PMC7374892

[65] Kim, K. A., Jeong, J. J., Yoo, S. Y. & Kim, D. H. Gut microbiota lipopolysaccharide accelerates inflamm-aging in mice. *BMC Microbiol* **16**, 9 (2016). 26772806 10.1186/s12866-016-0625-7 PMC4715324

[66] Chovatiya, R. & Medzhitov, R. Stress, inflammation, and defense of homeostasis. *Mol. Cell* **54**, 281–288 (2014). 24766892 10.1016/j.molcel.2014.03.030 PMC4048989

[67] Belanger, M., Rodrigues, P. H., Dunn, W. A. Jr & Progulske-Fox, A. Autophagy: a highway for Porphyromonas gingivalis in endothelial cells. *Autophagy* **2**, 165–170 (2006). 16874051 10.4161/auto.2828

[68] Diomede, F. et al. MyD88/ERK/NFkB pathways and pro-inflammatory cytokines release in periodontal ligament stem cells stimulated by Porphyromonas gingivalis. *Eur. J. Histochem* **61**, 2791 (2017). 28735521 10.4081/ejh.2017.2791 PMC5452629

[69] Hirasawa, M. & Kurita-Ochiai, T. Porphyromonas gingivalis induces apoptosis and autophagy via ER stress in human umbilical vein endothelial cells. *Mediators Inflamm.* **2018**, 1967506 (2018). 30150893 10.1155/2018/1967506 PMC6087591

[70] Inaba, H., Amano, A., Lamont, R. J., Murakami, Y. & Matsumoto-Nakano, M. Cell cycle arrest and apoptosis induced by porphyromonas gingivalis require Jun N-terminal protein kinase- and p53-mediated p38 activation in human trophoblasts. *Infect Immun* **86** (2018) 10.1128/IAI.00923-17 PMC5865039 29339463

[71] Kang, S., Dai, A., Wang, H. & Ding, P. H. Interaction between autophagy and porphyromonas gingivalis-induced inflammation. *Front Cell Infect. Microbiol* **12**, 892610 (2022). 35846745 10.3389/fcimb.2022.892610 PMC9283780

[72] Liang, D. Y. et al. Porphyromonas gingivalis infected macrophages upregulate CD36 expression via ERK/NF-κB pathway. *Cell Signal* **28**, 1292–1303 (2016). 27234131 10.1016/j.cellsig.2016.05.017

[73] Liu, J., Wang, Y. & Ouyang, X. Beyond toll-like receptors: Porphyromonas gingivalis induces IL-6, IL-8, and VCAM-1 expression through NOD-mediated NF-κB and ERK signaling pathways in periodontal fibroblasts. *Inflammation* **37**, 522–533 (2014). 24162780 10.1007/s10753-013-9766-0

[74] Lu, C., Chen, Z., Lu, H. & Zhao, K. Porphyromonas gingivalis lipopolysaccharide regulates cell proliferation, apoptosis, autophagy in esophageal squamous cell carcinoma via TLR4/MYD88/JNK pathway. *J. Clin. Biochem Nutr.* **74**, 213–220 (2024). 38799145 10.3164/jcbn.22-138 PMC11111472

[75] Meghil, M. M. et al. Disruption of immune homeostasis in human dendritic cells via regulation of autophagy and apoptosis by porphyromonas gingivalis. *Front Immunol.* **10**, 2286 (2019). 31608069 10.3389/fimmu.2019.02286 PMC6769118

[76] Kim, H.-J., Moon, C. M., Kang, J. L. & Park, E.-M. Aging effects on the diurnal patterns of gut microbial composition in male and female mice. *Korean J. Physiol. Pharm.* **25**, 575–583 (2021). 10.4196/kjpp.2021.25.6.575 PMC8552826 34697268

[77] Xiao, L. et al. A catalog of the mouse gut metagenome. *Nat. Biotechnol.* **33**, 1103–1108 (2015). 26414350 10.1038/nbt.3353

[78] Lozupone, C. A., Stombaugh, J. I., Gordon, J. I., Jansson, J. K. & Knight, R. Diversity, stability and resilience of the human gut microbiota. *Nature* **489**, 220–230 (2012). 22972295 10.1038/nature11550 PMC3577372

[79] Wade, W. G. Resilience of the oral microbiome. *Periodontol 2000* **86**, 113–122 (2021). 33690989 10.1111/prd.12365

[80] Popova, C., Dosseva, V. & Panov, V. Microbiology of Periodontal Diseases. *A Rev. Biotechnol. Biotechnol. Equip.* **27**, 3754–3759 (2014).

[81] Chen, W. A., Dou, Y., Fletcher, H. M. & Boskovic D. S. Local and systemic effects of porphyromonas gingivalis infection. *Microorganisms* **11** (2023) 10.3390/microorganisms11020470 PMC9963840 36838435

[82] Hajishengallis, G., Chavakis, T. & Lambris, J. D. Current understanding of periodontal disease pathogenesis and targets for host-modulation therapy. *Periodontol 2000* **84**, 14–34 (2020). 32844416 10.1111/prd.12331 PMC7457922

[83] Baker, P. J., Dixon, M., Evans, R. T. & Roopenian, D. C. Heterogeneity of Porphyromonas gingivalis strains in the induction of alveolar bone loss in mice. *Oral. Microbiol Immunol.* **15**, 27–32 (2000). 11155161 10.1034/j.1399-302x.2000.150105.x

[84] Baker, P. J., Dixon, M. & Roopenian, D. C. Genetic control of susceptibility to Porphyromonas gingivalis-induced alveolar bone loss in mice. *Infect. Immun.* **68**, 5864–5868 (2000). 10992496 10.1128/iai.68.10.5864-5868.2000 PMC101548

[85] Caporaso, J. G. et al. Ultra-high-throughput microbial community analysis on the Illumina HiSeq and MiSeq platforms. *ISME J.* **6**, 1621–1624 (2012). 22402401 10.1038/ismej.2012.8 PMC3400413

[86] Walters, W. Improved bacterial 16S rRNA Gene (V4 and V4-5) and fungal internal transcribed spacer marker gene primers for microbial community surveys. *mSystems* **1**, e00009-15 (2016). 27822518 10.1128/mSystems.00009-15 PMC5069754

[87] Bolyen, E. et al. Reproducible, interactive, scalable and extensible microbiome data science using QIIME 2. *Nat. Biotechnol.* **37**, 852–857 (2019). 31341288 10.1038/s41587-019-0209-9 PMC7015180

[88] Callahan, B. J. et al. DADA2: High-resolution sample inference from Illumina amplicon data. *Nat. Methods* **13**, 581–583 (2016). 27214047 10.1038/nmeth.3869 PMC4927377

[89] Quast, C. et al. The SILVA ribosomal RNA gene database project: improved data processing and web-based tools. *Nucleic Acids Res.* **41**, D590–D596 (2013). 23193283 10.1093/nar/gks1219 PMC3531112

[90] Friedman, J. & Alm, E. J. Inferring correlation networks from genomic survey data. *PLoS Comput Biol.* **8**, e1002687 (2012). 23028285 10.1371/journal.pcbi.1002687 PMC3447976

[91] Newman, M. E. Modularity and community structure in networks. *Proc. Natl. Acad. Sci. USA* **103**, 8577–8582 (2006). 16723398 10.1073/pnas.0601602103 PMC1482622

[92] Bastian, M., Heymann, S. & Jacomy, M. Gephi: An open source software for exploring and manipulating networks. *Proc. Int. AAAI Conf. Web Soc. Media* **3**, 361–362 (2009).

[93] Irie, K., Novince, C. M. & Darveau, R. P. Impact of the oral commensal flora on alveolar bone homeostasis. *J. Dent. Res* **93**, 801–806 (2014). 24935067 10.1177/0022034514540173 PMC4126224

[94] Matsuda, K., Haga-Tsujimura, M., Yoshie, S. & Shimomura-Kuroki, J. Characteristics of alveolar bone associated with physiological movement of molar in mice: a histological and histochemical study. *Odontology* **102**, 98–104 (2014). 23263522 10.1007/s10266-012-0093-y

[95] Amend, S. R., Valkenburg, K. C. & Pienta, K. J. Murine hind limb long bone dissection and bone marrow isolation. *J. Vis. Exp* **14**, 53936 (2016). 10.3791/53936 PMC4941920 27168390

[96] Koeberle, A., Shindou, H., Harayama, T., Yuki, K. & Shimizu, T. Polyunsaturated fatty acids are incorporated into maturating male mouse germ cells by lysophosphatidic acid acyltransferase 3. *FASEB J.* **26**, 169–180 (2012). 21968070 10.1096/fj.11-184879

[97] Koeberle, A. et al. Role of p38 mitogen-activated protein kinase in linking stearoyl-CoA desaturase-1 activity with endoplasmic reticulum homeostasis. *FASEB J.* **29**, 2439–2449 (2015). 25678624 10.1096/fj.14-268474

[98] Koeberle, A. et al. Arachidonoyl-phosphatidylcholine oscillates during the cell cycle and counteracts proliferation by suppressing Akt membrane binding. *Proc. Natl. Acad. Sci. USA* **110**, 2546–2551 (2013). 23359699 10.1073/pnas.1216182110 PMC3574958

