# Supplementary material for: Mediterranean diet component oleic acid decreases systemic impact of periodontal Porphyromonas gingivalis-infection in age: addressing role of stress resistance and microbiome
Source: NPJ Aging. 2025 Jun 28;11(1):54. doi: 10.1038/s41514-025-00248-7 (PMC12206234; doi:10.1038/s41514-025-00248-7)
Supplement: Supplementary file 1 — Revised Supplements [file 41514_2025_248_MOESM1_ESM.pdf]

Supplemental material

Supplementary Tables

Table S1 Diet content (Ssniff, Soest, Germany)

	U	**ND** 4.1 % fat	**PA-ED** 19.5 % fat	**OA-ED** 19.5 % fat
**Casein**	%	20.000	20.000	20.000
**Corn-starch, pre-gelatinized**	%	36.500	23.000	23.000
**Maltodextrin**	%	10.490	10.480	10.480
**Dextrose**	%	14.000	14.000	14.000
**Cellulose powder**	%	7.000	5.000	5.000
**Inulin**	%	2.000	2.000	2.000
**L-Cystine**	%	0.280	0.280	0.280
**Vitamin premix**	%	1.000	1.000	1.000
**Mineral & trace element premix**	%	4.500	4.500	4.500
**Choline Cl (50 %)**	%	0.200	0.200	0.200
**Dye [yellow/green/red/blue/M]**	%	0.030	0.040	0.030
**Palm oil**	%	----	9.300	----
**Palmitate ethyl ester ≥97 %**	%	----	9.300	----
**HO Sunflower oil**	%	3.100	----	18.600
**Soybean oil**	%	0.900	0.900	0.900
**Proximate contents**				
**Crude protein**	%	17.7	17.7	17.7
**Crude fat**	%	4.1	9.5	9.5
**Crude fiber**	%	8.8	6.8	6.8
**Crude ash**	%	4.0	4.0	4.0
**Starch**	%	35.1	22.1	22.1
**Dextrin**	%	10.	10.4	10.4
**Sugar**	%	13.9	13.9	13.9
**NfE (carbohydrates)**	%	60.4	47.4	47.4
**Energy (Atwater)**	MJ/kg	14.6	18.3	18.3
**kcal% Protein**		20	16	16
**kcal% Fat**		11	40	40
**kcal% Carbohydrates**		69	44	44

**Fatty acids % in the diet**				
**C 14:0**		0.01	0.10	0.01
**C 16:0**		0.25	12.52	0.83
**C 18:0**		0.13	0.47	0.59
**C 20:0**		0.01	0.04	0.05
**C 16:1**		0.01	0.02	0.02
**C 18:1**		2.85	3.92	15.87
**C 20:1**		0.01	0.01	0.06
**C 18:2**		0.66	1.32	1.62
**C 18:3**		0.03	0.07	0.08

Normal standard diet (**ND**), palmitic acid (C16:0) enriched diet (**PA-ED**), oleic acid (C18:1) enriched diet (**OA-ED**)

Table S2 Illumina adapter constructs for 16S rRNA amplicon sequencing

CONSTRUCT	ELEMENTS	SEQUENCE
**FORWARD**	5' - Illumina Adapter	AATGATACGGCGACCACCGAGATCTACACGCT
	Golay barcode	NNNNNNNNNNNN
	Forward primer pad	TATGGTAATT
	Forward linker	GG
	Forward primer (F515)	GTGYCAGCMGCCGCGGTAA
**REVERSE**	3' - Illumina Adapter	CAAGCAGAAGACGGCATACGAGAT
	Reverse primer pad	AGTCAGCCAG
	Reverse linker	CC
	Reverse primer (R806)	GGACTACNVGGGTWTCTAAT

Supplementary Figures

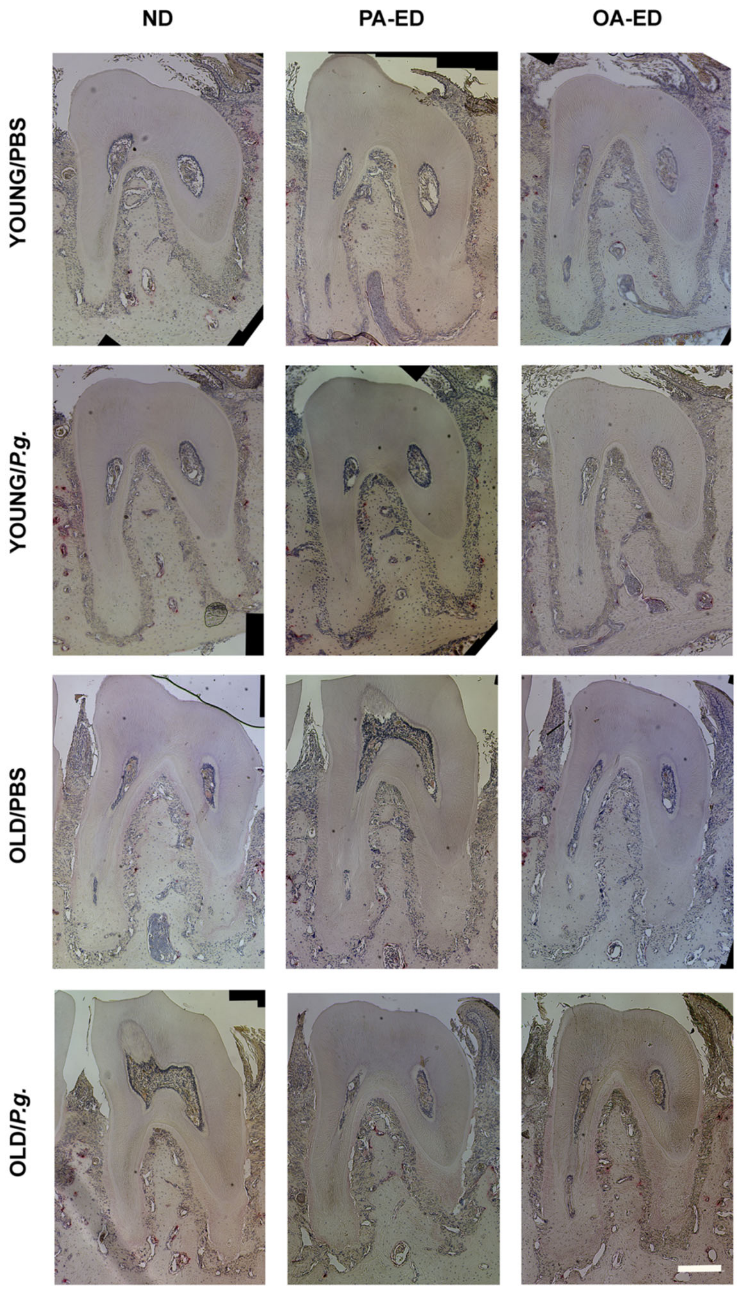

Figure S1 Immunohistochemical images as a basis for the histomorphometric evaluation of jawbone quality as a function of diet, age and infection. Young (5 weeks) and aged (≥73 weeks) male C57BL6Rj received normal diet (ND), palmitic acid enriched diet (PA-ED) or oleic acid. Animals were orally inoculated with *Porphyromonas gingivalis* (P.g.) and compared to mock- (PBS)-infected controls. Mandibular bone sections of young and old control or infection animals were TRAP-stained after 16 weeks of dietary intervention. Sections were stained for TRAP (depicted in red) and counterstained with Meyer's Hemalaun (blue). **Scale bar: 200 µm**

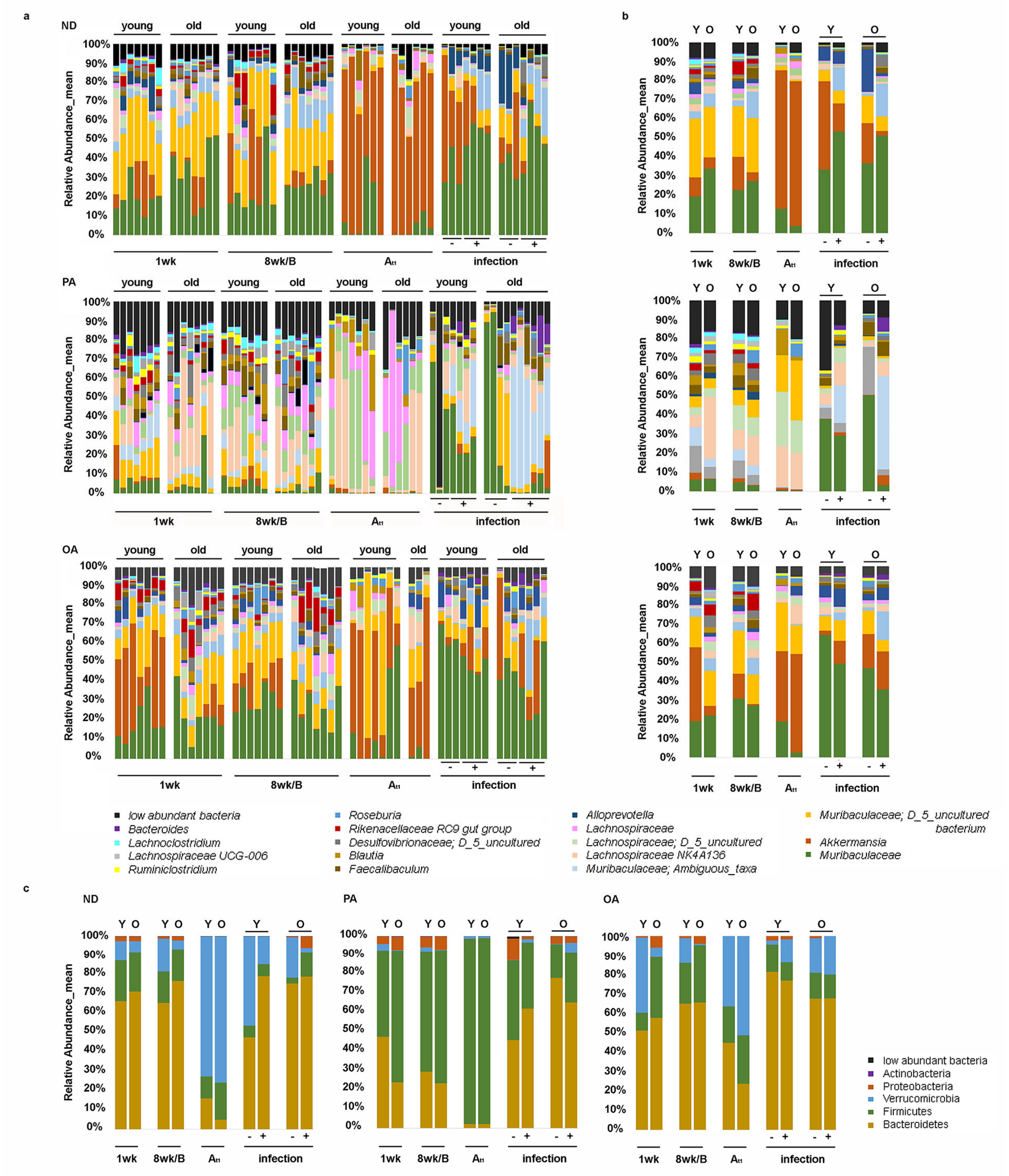

Figure S2 Analysis of quantitative and compositional changes in the murine microbiome in the course of different interventions. Male C57BL6Rj received normal diet (ND), palmitic acid enriched diet (PA-ED) or oleic acid enriched diet (OA-ED) for a total of 16 weeks. Animals received an antibiotic treatment in week 9 prior to onset of 5-week-inoculation with *Porphyromonas gingivalis* (*P.g.*). Sampling was performed after 1 week of DI (1wk), after 8 weeks of DI/before antibiosis (8wk/BAt$_1$), after antibiosis, after *P.g,* or CTRL-inoculation. Shown are **a** the taxonomic changes of single animals and **b** summarized to bar per group **c** and changes on phylum level during dietary intervention, antibiotic treatment and *P.gingivalis*-inoculation. DI- dietary intervention, ND- normal diet, PA-ED -palmitic acid enriched diet, OA-ED- oleic acid enriched diet

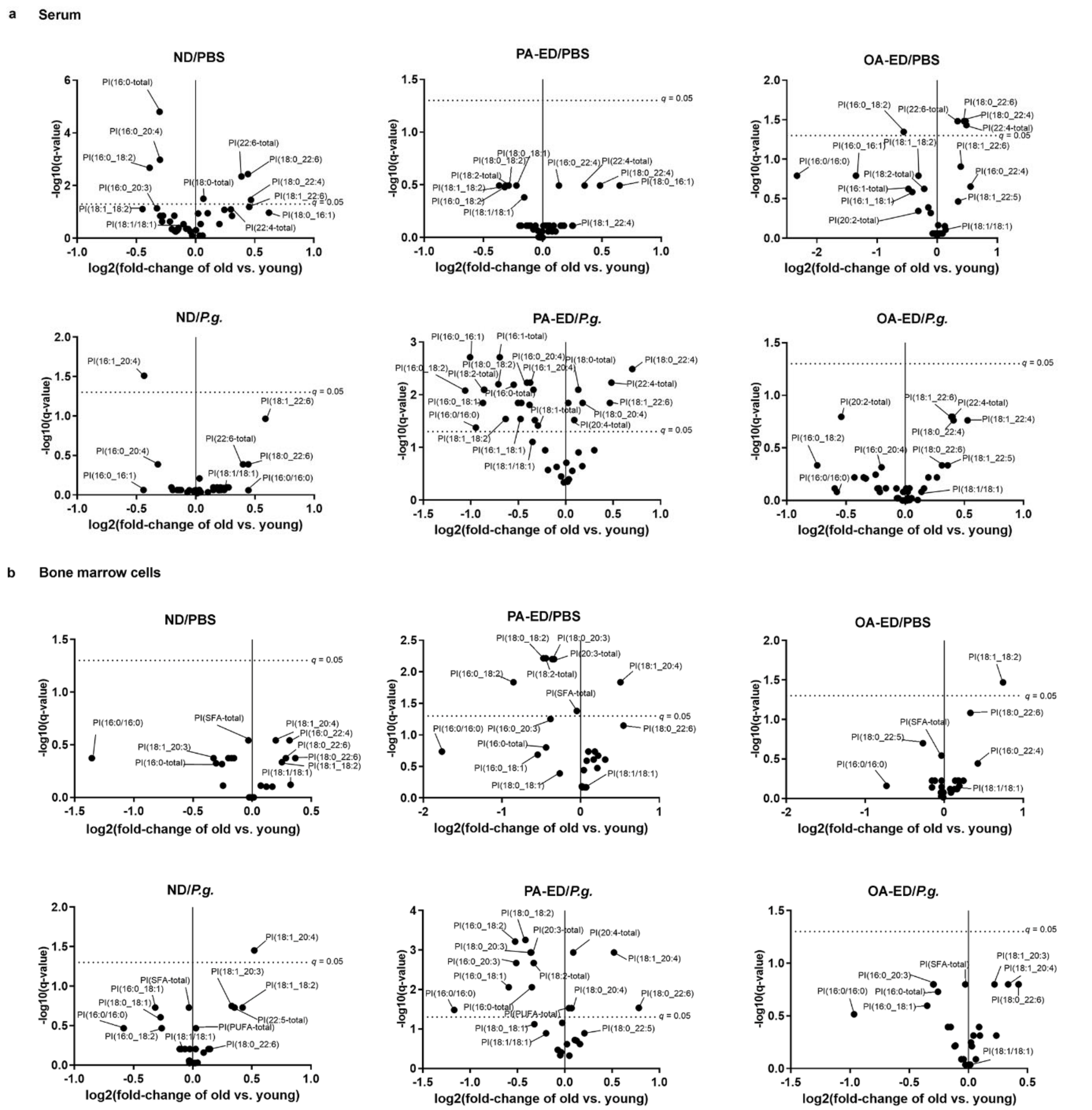

Figure S3 Effect of age and infection on the PI profile of mouse serum and murine bone marrow cells. Old (> 20 months) and young mice (21 weeks) have been treated with different diets (ND, normal diet; PA, palmitic acid enriched diet; OA, oleic acid enriched diet) for **a** 16 weeks and infected with PBS or *Porphyromonas gingivalis* (PG) for 5 weeks. Mouse serum was collected, extracted and PI species were analyzed by UPLC-MS/MS. **b** Dietary intervention was performed as described in panel A, but only for up to 9 weeks. 2.5×10^6 bone marrow cells were collected after sacrifice, seeded for 2 days and infected *ex vivo* with PBS or *P.gingivalis* (P.g.) for 6 h. Cell pellets were collected, extracted and PI species were analyzed by UPLC-MS/MS. **a**, **b** Volcano-plots showing the fold change in the proportion of PI species and subclasses (% of total PI) and the corresponding *q*-value of the comparison between old against young mice with the same treatment. The label 'fatty acid-total' defines the overall proportion of the indicated fatty acid in PI. *q*-Values between old and young mice of the same treatment were determined by multiple unpaired *t*-tests with 5% false discovery rate (FDR). Data are given as mean ± SEM, from *n* = 3-6 mice. SFA – saturated fatty acid, PUFA –polyunsaturated fatty acid, ND- normal diet, PA-ED -palmitic acid enriched diet, OA-ED- oleic acid enriched diet, ND- normal diet, PA-ED -palmitic acid enriched diet, OA-ED- oleic acid enriched diet
